# Vitamin D, C-Reactive Protein, and Increased Fall Risk: A Genetic Epidemiological Study

**DOI:** 10.3390/nu17010038

**Published:** 2024-12-26

**Authors:** Joshua P. Sutherland, Ang Zhou, Elina Hyppönen

**Affiliations:** 1 Australian Centre for Precision Health, Unit of Clinical and Health Sciences, University of South Australia, Adelaide, SA 5000, Australia; joshua.sutherland@mymail.unisa.edu.au; 2 South Australian Health and Medical Research Institute, Adelaide, SA 5000, Australia; 3 Medical Research Council Biostatistics Unit, University of Cambridge, Cambridge CB2 0SR, UK; ang.zhou@mrc-bsu.cam.ac.uk

**Keywords:** falls, motor function, vitamin D, mendelian randomization analysis

## Abstract

*Background*: Falls are a major public health concern. Daily vitamin D supplementation is a proposed fall prevention strategy; however, safety concerns have arisen from some clinical trials showing increased fall risk when using higher vitamin D dosing methods. The relationship between vitamin D and falls may be influenced by factors, such as inflammation, which can alter the balance of essential nutrients like vitamin D and retinol, potentially affecting motor function. We use a genetic epidemiological approach to explore the association of inflammation, vitamin D, and fall risk. *Methods:* We included 307,082 UK Biobank participants and conducted observational and Mendelian randomization (MR) analyses to investigate associations between 25-hydroxyvitamin D [25(OH)D] and fall risk, with analyses including restriction to participants who had fallen and had inflammation as defined by CRP ≥ 5 mg/L. *Results:* In the observational analysis, CRP was associated with a higher (per 5 mg/L CRP increase OR = 1.06, 95% CI 1.05–1.07) and 25(OH)D with a lower odds of falls. The association between 25(OH)D concentrations and fall risk was non-linear (*p* < 0.001), reflecting a plateauing of the association at higher concentrations. There was an interaction between 25(OH)D and CRP on their association with the odds of falls (*p* = 0.009). In participants with CRP ≥ 5 mg/L, the association was U-shaped, and the fall risk was elevated for both 25(OH)D < 25 nmol/L and ≥ 100 nmol/L (*p* < 0.004). The association between high 25(OH)D and falls was most pronounced for participants with CRP ≥ 20 mg/L (≥ 100 nmol/L vs. 50–74.99 nmol/L: OR = 2.40, 95% CI, 1.50–3.86). Genetically predicted higher 25(OH)D was not associated with fall risk in the overall population, but a suggestive association with fall risk was seen in participants who had fallen and had CRP > 20 mg/L (926 cases; OR = 1.20, 95% CI, 1.00–1.44). *Conclusions:* Our study suggests that inflammation might modify the vitamin D and fall risk relationship. Both low and high 25(OH)D levels are associated with more falls in individuals with chronic inflammation, with supporting evidence seen in both observational and MR analyses. This may provide insight into the increased fall risk following high-dose vitamin D supplementation in clinical trials, warranting further research.

## 1. Introduction

Falls represent a significant public health concern, leading to considerable morbidity, mortality, and economic burden on healthcare systems globally [1,2]. Research suggests that a single fall prevention program could prevent upward of 40,000 falls per year in the US and avoid approximately 100 to 400 million spent annually on medical expenses [3]. In people aged 65 and above, falls are the leading cause of injury [4], and therefore, identifying factors that mitigate fall risk in older people is crucial. Daily vitamin D supplementation programs, aimed at redressing vitamin D insufficiency, have been highlighted as one avenue for fall prevention [3]. This is due to the relationship between vitamin D deficiency and increased fall risk [5], as well as the reduction in falls seen in clinical trials using daily dosing of vitamin D3 at 700 to 2000 IU/day, and especially when given to the vitamin D deficient individuals [6,7].

However, vitamin D and fall space are not without controversy. The safety of vitamin D supplementation has been questioned after several clinical trials reported an increased fall risk among the elderly following annual/monthly bolus dosing or high daily doses (>3200 IU [8,9]). A study by Sanders et al. (2010) found an increased fall risk after administering a single oral dose of 500,000 IU of vitamin D3 [10]. Similar outcomes, in primary and subgroup analyses, were observed in other studies using monthly doses (60,000 IU to 100,000 IU) [11,12,13]. However, some notable trials, like the ViDA trial, did not report an increased fall risk despite using similar dosages (100,000 IU monthly) [14], and meta-analyses on vitamin D supplementation have shown conflicting results [8,15]. Importantly, the Sanders study remains unique, as no other motor function-related clinical trial has administered a comparably high single oral dose to an almost entirely vitamin D-replete population [10].

One aspect that might link vitamin D and the risk of falls, and which may explain the observed inconsistent associations, is inflammation. Inflammation (commonly indexed by elevated C-reactive protein (CRP) concentrations [16]) is associated with a higher rate of falls [17,18,19], particularly in the elderly [20,21]. Research suggests that inflammation may impact fall-related pathology through both direct and indirect mechanisms, including impairing neurological function, increasing muscle/joint weakness and pain, and exacerbating comorbid conditions associated with falls, such as depression and frailty [17,18,19,20,21]. Inflammation can also impact retinol status, which is important for the current context as retinol has a direct competitive relationship with vitamin D [22,23,24], and it plays an important role in the nervous system in supporting motor function regulation [25,26]. Elevated CRP concentrations are associated with lower retinol levels, and for example, a modestly elevated CRP (≥5 mg/L) has been associated with 25% lower retinol levels compared to those with CRP < 5 mg/L [27]. Given that retinol and vitamin D have a competitive relationship, inflammation could disrupt the balance between these two steroid hormones.

Therefore, in this genetic epidemiological study, we use data from the UK Biobank to safely explore the interrelationships between higher 25(OH)D, CRP, and the risk of falls.

## 2. Methods

We used information from the UK Biobank, which is a prospective cohort that consists of 502,316 individuals aged between 37 and 73 years, assessed from March 2006 to July 2010 in England, Scotland, and Wales [28]. The participants provided biological samples and participated in survey questionnaires and physical assessments. Analysis in our study was limited to unrelated participants of European ancestry, with MR analyses containing 307,082 individuals, upon restriction to the availability of serum 25(OH)D concentrations. A full overview of exclusion criteria, covariate missingness, composition of models—and the outcome variable case and control numbers therein—can be found in the Supplementary Materials (Figure S1). The UK Biobank was approved by the National Information Governance Board for Health and Social Care and the North West Multicenter Research Ethics Committee (11/NW/0382), and informed consent was obtained from all participants. The current study was conducted under UK Biobank project number 20175.

Baseline serum 25(OH)D concentrations were measured using the LIAISON XL 25(OH)D assay from DiaSorin (Stillwater, OK, USA), and the details of the measurement procedure can be found in the Supplementary Materials, page 3. For models using the categorical 25(OH)D, we divided the participants into those with 25(OH)D < 25 nmol/L (reflecting a low vitamin D status [5]), and then into subsequent categorical iterations of 25 nmol/L—25–49.9 nmol/L, 50–74.9 nmol/L, 75–99 nmol/L, and ≥100 nmol/L, using participants with average/normal levels (50–74.9 nmol/L) as the reference group. Concentrations of serum CRP were measured using high sensitivity analysis via an immunoturbidimetric assay, performed on a Beckman Coulter AU5800 instrument (Brea, CA, USA), with an analytical range of 0.08 to 79.96 mg/L [29]. Information for the fall outcome was acquired at baseline via a self-reported questionnaire, containing the question “In the last year have you had any falls”, which in our study was coded as a ‘yes’ or ‘no’ variable, excluding those who preferred not to answer. We considered 5 mg/L as the initial CRP threshold [30], as this has been shown to be associated with ~25% retinol reduction [27]. As retinol deficiency could be expected to be more severe the stronger the inflammation, we also categorized CRP using thresholds defined by 5 mg/L increments up to where enough data to facilitate analysis were retained (CRP ≥ 10 mg/L, ≥15 mg/L, and ≥20 mg/L; ≥20 mg/retaining 940 cases).

Information on potential confounders was obtained from self-reported touchscreen questionnaires (physical activity, smoking, alcohol, age, and sex), physical assessments (height and weight for BMI calculation), and residential address data (Townsend deprivation index). These covariates were used in model adjustments for the phenotypic analyses and are detailed in the Supplementary Materials (pages 3 to 4, and Figure S2).

For the genetic data, the UK Biobank used the Axiom array, which was designed specifically for the UK Biobank and includes approximately 820,000 genetic markers, both common and rare variants [31]. The UK Biobank conducted quality assurance processes to ensure the accuracy and reliability of their genotyping data relating to sample, genotyping, and imputation quality. These processes were described in detail in the genotyping and quality control report and elsewhere [32,33]. For our MR analysis, a weighted genetic score was developed by compiling 35 common autosomal SNPs, which were identified from a genome-wide association study conducted on measured 25(OH)D concentration [34,35] and independently validated in the SUNLIGHT consortium [36]. Further details on the development of the vitamin D genetic score and the selection of the variants utilized can be found in the Supplementary Materials (including Figure S3 and Table S1).

### 2.1. Statistical Methods

*Phenotypic analyses*: We used linear regression to investigate 25(OH)D association with log-transformed CRP, and logistic regression to assess the CRP–fall and 25(OH)D–fall associations. We examined if the 25(OH)D–fall association varied by CRP by including an interaction term [CRP × 25(OH)D] in the model. In the presence of interaction, we stratified the models by levels of CRP. To investigate whether the association of 25(OH)D with fall risk is similar for falls which occur in the presence of inflammation, we also conducted analyses defining the outcome of falls based on whether it concurred with elevated CRP concentrations at various thresholds (≥5/10/15/20 mg/L). In the simple model, we adjusted for age, sex, and assessment center. For analyses with 25(OH)D concentrations as the exposure only, we also controlled for nuisance factors which could affect the measured 25(OH)D concentrations (blood sampling month, fasting time before sample acquisition, and aliquot). In the full model, we further adjusted for BMI, physical activity (low, moderate, and high), alcohol (daily or almost daily, once or twice a week, three or four times a week, one to three times a month, special occasions only, and never), smoking (non-smokers, former smokers, and current smokers), education (none, national vocational qualification/certificate of secondary education/A-levels, and degree/professional), and Townsend deprivation index (quartile division of index: least to most deprivation).

*Mendelian randomization analyses*: We examined genetic evidence for causality in the association of 25(OH)D and falls using the MR approach (Supplementary Figure S4). Our primary MR analyses were conducted using the weighted genetic score constructed from the 35 SNPs associated with 25(OH)D, where the weights were based on estimates of SNP-25(OH)D association from the SUNLIGHT Consortium meta-analyses [36]. We conducted sensitivity analyses applying complementary two-sample MR methods, notably IVW, MR-PRESSO, Weighted median, Weighted mode, and MR-Egger. Further details are provided in the Supplementary Materials (pages 4 to 9).

Causal inference in Mendelian Randomization (MR) analysis relies on three assumptions [37] (Figure S2): (1) Genetic score for vitamin D associates with measured 25(OH)D concentrations. (2) Genetic score for vitamin D has no direct impact on falls. (3) Genetic score for vitamin D does not associate with confounders of 25(OH)D and falls. To assess the first assumption, we examined the association between the genetic score and 25(OH)D in the UK Biobank (Figure S5). Potential horizontal pleiotropy, where the genetic instrument affects outcomes through other pathways, can violate the second and/or third assumptions. To identify horizontal pleiotropy, we assessed the genetic score’s association with potential confounders (Table S2). We also conducted a leave-block-out analysis, where we excluded variants which had been suggested to have effects also on additional traits (Supplementary Materials page 9, Tables S3 and S4) [38]. We also conducted sensitivity analyses using a broader 122 SNP version of the genetic score (Table S1).

R, version 3.6.1 was used for linear MR sensitivity analyses (‘TwoSampleMR’ and ‘MRPRESSO’ packages), and STATA, version 14.1 (Stata-Corp LP, College Station, TX, USA) was used for all other analyses.

### 2.2. Role of Funding Source

The study was supported by the National Health and Medical Research Council (NHMRC), grant number 11123603. The Joshua P Sutherland studentship is funded by the Australian Research Training Program Scholarship. Elina Hypponen is funded by the National Health and Medical Research Council (NHMRC) leadership award (GNT 2025349). Neither funding source had any role in this study’s design, conduct or reporting.

## 3. Results

As shown in Table 1 (N = 307,082), the average serum concentration of CRP and 25(OH)D in participants was 1.38 mg/L (geometric mean) and 49.8 nmol/L, respectively. CRP levels were higher in females vs. males, those aged ≥ 60 years vs. <60 years, current smokers, and in those with the highest BMI. Compared to others, average levels of 25(OH)D were lower in those with the highest BMI, the lowest physical activity, the northernly located, and current smokers. Falls were more commonly reported by those older than age sixty compared to younger participants, by females compared to males, and by those with the most compared to least socioeconomic deprivation. The prevalence of falls in individuals with high CRP (reported at ≥5 mg/L and ≥20 mg/L, for the purpose of Table 1) was particularly evident in the obese, current smokers, the most socioeconomically deprived, and the non-educated.

### Phenotypic Analysis

The association between CRP and 25(OH)D was non-linear (*p* < 0.001), and in the fully adjusted analyses, log-transformed CRP levels were higher at both lower and higher 25(OH)D categories, relative to the 25(OH)D reference category of 50–74.99 nmol/L (Table 2). CRP was associated with an increased fall risk (P-non-linearity < 0.001), showing a positive association that plateaued at higher concentrations (Table S5). We observed an inverse cross-sectional association between 25(OH)D concentrations and fall risk, with evidence of non-linearity reflecting a plateauing of the association at higher concentrations (*p* < 0.001) (Table 3). However, we also observed evidence for the interaction, suggesting that the 25(OH)D-fall relationship differed by CRP levels (*p* = 0.009). To explore this interaction, we conducted stratified analyses of the 25(OH)D-fall risk relationship above and below the different CRP thresholds (≥ and <5/10/15/20 mg/L). Below these respective CRP thresholds, we observed an inverse association between 25(OH)D and fall risk (Table S6). Above these thresholds, the inverse association remained for CRP ≥ 5 mg/L and ≥10 mg/L, but with greater uncertainty for higher thresholds (e.g., 838 cases and 2421 controls for CRP ≥ 20 mg/L) (Table S6).

**Figure 1:**
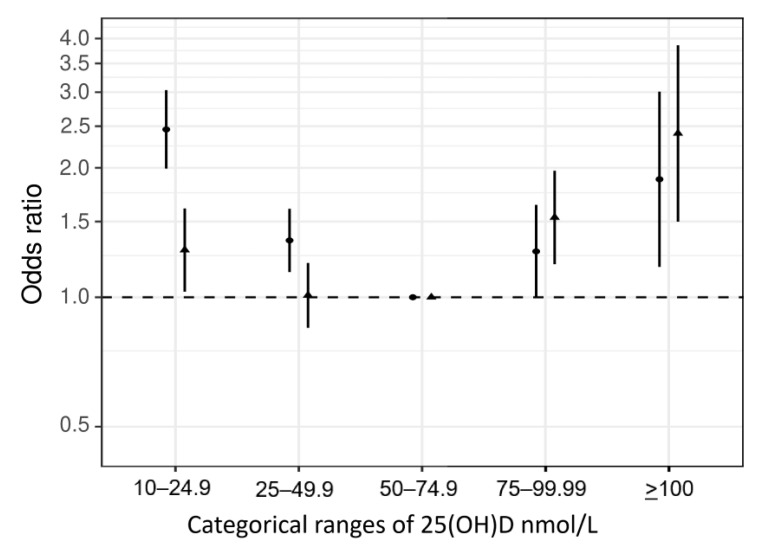
Phenotypic analyses for the association of measured 25(OH)D and falls in the UK Biobank, with the outcome restricted to falls concurring with C-reactive protein > 20 mg/L. First line = simple ⬤, second line = adjusted ▲. Reference category: 50–74.99 nmol/L. For *p* values, see Table 3. Simple models were adjusted for sex, age, assessment center, and nuisance factors that could affect 25(OH)D serum measurements, including the month in which the blood sample was taken, fasting time before the blood sample was taken, and sample aliquots for measurement; fully adjusted models were additionally adjusted for educational status, Townsend deprivation index, body mass index, physical activity, alcohol, and smoking.

We then repeated the initial 25(OH)D-fall analysis, defining cases only as those who fell and had CRP above the thresholds of ≥5/10/15/20 mg/L (e.g., 838 cases and 239,322 controls for CRP ≥ 20 mg/L) (Table S7). In these analyses, we observed strong evidence for non-linearity (*p* = 0.00009), and the highest odds of falls co-occurring with inflammation (CRP > 20 mg/L) was seen for those with the highest 25(OH)D levels, while a higher risk was also confirmed for participants with 25(OH)D < 25 nmol/L (≥100 nmol/L vs. 50–74.99 nmol/L: OR = 2.40, 95% CI, 1.50–3.86; and <25 nmol/L vs. 50–74.99 nmol/L: OR = 1.29, 95% CI, 1.03–1.61) (Figure 1). The non-linear association between 25(OH)D and increased fall risk was consistent and significant across the CRP thresholds, being the more pronounced, the higher the CRP (Table 3).

For the sensitivity analyses to explore the association between 25(OH)D and fall risk in the context of high inflammation, we repeated the categorical analysis in a rheumatoid arthritis patient population (typically characterized by higher inflammation). In this setting, the association between both lower and higher 25(OH)D and increased fall risk was consistent and significant among those who fell and had rheumatoid arthritis (915 cases, 238,968 controls) (≥100 nmol/L vs. 50–74.99 nmol/L: OR = 1.64, 95%, CI, 1.00–2.69, *p* < 0.05; and <25 nmol/L vs. 50–74.99 nmol/L: OR = 1.28, 95%, CI, 1.03–1.60, *p* = 0.03) (see Table S8). To explore the association in the context where inflammation is not present, we repeated the categorical analysis by restricting it to falls occurring below <5 mg/L CRP, and this reflected an association with fall risk in the deficiency categories (Table S9).

**Figure 2:**
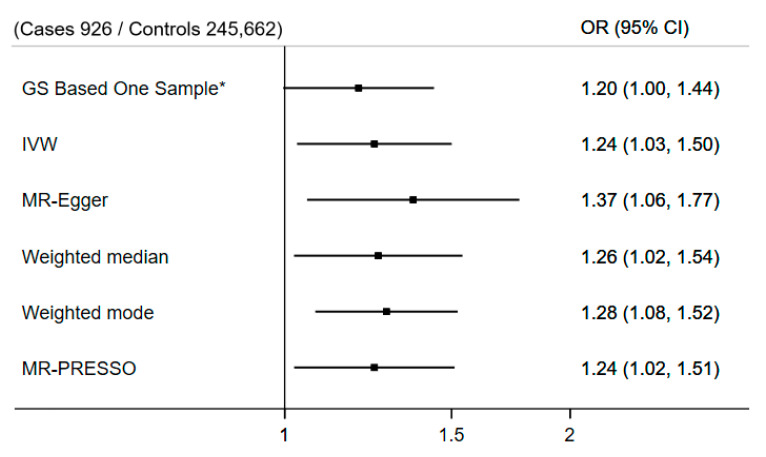
Mendelian randomization sensitivity analyses of genetically predicted 25(OH)D and falls concurring with C-reactive protein > 20 mg/L in the UK biobank. The Genetic Score (GS)-based one-sample approach is presented for comparison purposes * (all other MR approaches presented are two-sample). In the GS-based one-sample approach, the weight of SNPs are based on independent SNP-25(OH)D beta coefficients from the Sunlight Consortium [36]. Genetic analysis adjusted for age, sex, assessment center, SNP array, principal components 1–40, and nuisance factors which could affect serum 25(OH)D measurements, including the month in which the blood sample was taken, fasting time before the blood sample was taken, and sample aliquots for measurement. MR PRESSO outlier/s = N/A.

*Genetic analysis—Mendelian randomization*: We next conducted MR analyses to first assess the association of genetically predicted 25(OH)D and fall outcomes in the general population. In linear MR analyses, there was no evidence for an association between genetically predicted 25(OH)D and falls using the unrestricted outcome sample (fall cases n = 59,303) (per 10 mg/L increase in 25(OH)D, OR = 1.01, 95% CI 0.99–1.04, *p* = 0.2). However, the same MR analysis but repeated with fall cases reflecting those who had fallen and had CRP above thresholds ≥5/10/15/20 mg/L (e.g., 926 cases for CRP ≥ 20 mg/L) provided tentative evidence of a linear association where fall risk increased along with higher 25(OH)D concentrations. This pattern became more pronounced with each threshold increase, with nominal evidence at CRP > 20 mg/L (per 10 mg/L increase in 25(OH)D, OR = 1.20, 95% CI, 1.00–1.44, *p* = 0.05). Sensitivity analyses using the CRP > 20 mg/L threshold generated similar results when using the 122 SNP version of the genetic score (Table S3), across several pleiotropy robust MR approaches (Figure 2), and in the leave-block-out analyses (Table S3).

## 4. Discussion

In this paper, we explored the relationship between 25(OH)D and fall risk in the context of inflammation, given that inflammation may be a setting where motor function supporting factors, such as retinol [22,39,40], could be disrupted by higher vitamin D. We show that the association between vitamin D status measured by 25(OH)D and the inflammatory marker CRP is likely complex, with more inflammation observed both for participants with the lowest and the highest 25(OH)D concentrations. We also show that the association between 25(OH)D concentrations and the risk of falls appears to be inverse overall, with the highest risks seen for those with the lowest concentrations. However, for participants with the highest 25(OH)D concentrations, the risk of falling is higher if they also had evidence for high inflammation, with this finding supported by genetic evidence showing a positive association between higher 25(OH)D and inflammation-related fall risk. Overall, our analyses suggest that the association of 25(OH)D and the risk of falls depends on the presence of inflammation, which is compatible with the notion that inflammation suppresses the retinoid system [27]—an important neurological regulator of motor function [25,26], and a system in competition with vitamin D [22,23,24].

Previous studies examining vitamin D status and fall risk have generally observed lower 25(OH)D levels (<50 nmol/L), associating this with an increased risk of falls, particularly among older adults [41,42,43,44,45]—and this aligns with vitamin D trials that show a reduction in fall risk arising particularly in those who are deficient [7]. The mechanisms proposed to account for the fall risk and vitamin D insufficiency relationship typically focus on factors, including impaired muscle function due to vitamin D receptor-mediated effects on muscle cells [46], reduced bone integrity from secondary hyperparathyroidism, leading to increased bone resorption [47], and vitamin D deficiency-induced inflammation that may exacerbate neurological a neuromuscular dysfunction [48]. While these proposed mechanisms address fall risk associated with chronic vitamin D insufficiency, our study extends this consideration to factors that may be relevant in the context of transiently higher vitamin D.

Clinical trials have shown mixed results regarding the protective effects of vitamin D against falls [8,15,49]; however, findings generally suggest that—where benefits are seen—they arise from smaller daily dosing (<2000 IU vitamin D3), and they do not extend to those with sufficient vitamin D levels [6,7]. Those studies that have reported increased fall risk (in primary or subgroup analyses) with annual (500,000 IU) [10], monthly (60,000 IU to 100,000 IU) [11,12,13], or high daily intakes (>3200 IU) of vitamin D3 [8,9] suggest the presence of a threshold beyond which additional vitamin D may no longer prevent falls, and could even pose a transient increase in fall risk. Inconsistencies notably include the ViDA trial, which used 100,000 IU monthly and did not report adverse effects [14]. However, the study by Sanders et al. (2010)—which reported an increased risk of falls—is notable, in that no other motor function-related study has administered a single oral dose of 500,000 IU vitamin D3 to a largely vitamin D-replete population [10]. The study by Latham et al. (2003)—which did not report increased fall risk—is the closest related trial, having used a single 300,000 IU dose of oral vitamin D3 [50]. However, the prevalence of deficiency was notably higher in the Latham trial [30% under 30 nmol/L 25(OH)D], while in the trial of Sanders et al., only 3% had 25(OH)D under 25 nmol/L [10,50]. Differences in baseline vitamin D repletion across the studied populations may also be a critical factor that biases meta-analyses of these findings.

One explanation that potentially links fall risk with both lower and higher 25(OH)D levels is inflammation, albeit through distinct mechanisms. Unlike the potential fall risk posed by inadequate VDR stimulation of anti-inflammatory immunomodulatory cells, which can occur in the state of vitamin D deficiency [51], the presence of inflammation in conjunction with higher vitamin D levels may shift the balance of steroid hormone competition [22,23,24]. This may affect both retinol’s anti-inflammatory role [52], and its neurological-based regulation of motor function [25,26]. The competitive relationship between vitamin D and retinol is mechanistically explainable at the level of heterodimer synthesis and retinoic acid response elements (RARE) binding. The primary nuclear receptors that RA binds to are the retinoic acid receptor (RAR) and retinoid X receptor (RXR) [53], with vitamin D receptor (VDR)-RXR heterodimers also capable of binding to RARE. In this context, the VDR-RXR produces a repressive heterodimer species that can suppress RARE-mediated gene expression that would otherwise occur from RAR-RXR stimulation [54,55,56].

Vitamin D’s role in inflammation has been widely studied [51]; however, the relationship between vitamin D and CRP presents complex and sometimes contradictory findings [57,58,59,60,61,62,63]. Several observational studies have reported inverse associations [57,58,59], supporting the notion that higher vitamin D might reduce systemic inflammation. While a 2018 meta-analysis of trial data showed no overall impact of vitamin D supplementation on CRP, the included studies that did show a lowering of CRP were largely using lower vitamin D3 doses (400–7000 IU) or bolus doses given to vitamin D deficient cohorts [63]. Meanwhile, a 2024 meta-analysis found a reduction in CRP levels in post-menopausal women from ≥3 months of daily vitamin D3 1000 IU [62]. These findings suggest that the vitamin D and CRP relationship may be another complex physiological scenario dependent on repletion status and dosing methods, and where more vitamin D—beyond addressing deficiency—may not necessarily be better.

The null MR findings—derived from analyzing the full sample of falls—suggest that the increased risk of falls associated with higher 25(OH)D is not present in normal physiological conditions, where natural fluctuations of 25(OH)D occur within typical serum level ranges and are likely in balance with other dietary derived steroid hormones. However, given that we saw a genetic association between higher 25(OH)D and increased fall risk in those who fell and had CRP ≥ 20 mg/L, this could suggest the potential for clinically relevant/discernible outcomes arising from disruption to steroid hormone homeostasis—whether that be derived from dietary retinol deficiency, retinol impairment, bolus dosing of vitamin D, or other inflammatory-related effects on steroid hormone function. While a mechanistic relationship between retinol and vitamin D was considered as a theoretical basis linking distinct observations related to vitamin D, inflammation, and falls, further research will need to elucidate if these proposed mechanisms underlie our findings, and if the phenomenon might occur in response to other forms of interaction between inflammation, vitamin D, and motor function regulation. Further studies using animal models (e.g., murine or drosophila melanogaster [64]) could explore the mechanistic insights raised here, and more effectively explore the potential interactions between inflammation, vitamin D and retinol [27].

Bolus dosing of vitamin D has been safety assessed by multiple government regulators around the world prior to implementation in medical settings [65,66]. However, an increased risk of falling is likely related to factors beyond what safety trials have typically been primed to assess. While the retinol competition could potentially explain the higher risk of falls observed in these trials, there are other possible mechanisms. For example, high-dose oral vitamin D is sometimes used to induce vascular calcification in animal models [67]. There are also multiple recorded cases in humans where very high to acutely toxic intakes of vitamin D supplementation, given by error, have resulted in irreversible tissue calcification [68,69]. However, there is no reported indication that the increased falling (associated with bolus dosing of vitamin D) had concurred with calcification tissue injury, which is usually notable, chronic, and irreversible. Other plausible mechanisms could be those which influence vestibular-related tissues/function, although, to our knowledge, no related associations have been reported in the bolus dosing clinical trials [9,10,11,12,13]. While we do not know if the affected clinical trial participants (who reacted poorly to large dose supplementation [9,10,11,12,13]) had inflammation, our results could suggest that inflammation plays a broader role in high vitamin D-related fall risk scenarios.

### Strengths and Limitations

This study has made pragmatic use of available resources to safely and ethically assess metabolic steps that may underlie a phenomenon that has become challenging to navigate via conventional clinical trial pathways. Our analyses used large-scale data obtained from the UK Biobank, with comprehensive analyses to address and assess associations, at both high and low concentrations. Importantly, results from the MR analyses on 25(OH)D associations and inflammation-related risk(s) of fall were consistent across different approaches and supported the findings from observational analyses. While we cannot emulate the same clinically relevant estimations regarding fall risk that have emerged from clinical trials, our study, within the limits of data availability, provides support for potential underlying mechanisms that are worthy of consideration and further exploration. However, our study also has limitations, and while the UK Biobank provided the size necessary to detect the phenomenon in a non-clinical trial environment, it lacked measures for some key biomarkers, such as specific inflammatory factors and retinol (which was only indirectly included via a subgroup sample of 24 h recall dietary retinol data, which was not validated against serum retinol). While our analyses assessing fall risk at higher CRP thresholds suggest a complex interaction between inflammation and the vitamin D-fall association, we acknowledge that smaller sample sizes in these stratified subgroups may have affected or limited the precision of the estimates. However, the range of methods employed for these subgroup analyses, including MR, collectively supports the presence of a potential biological phenomenon underlying this association.

Further limitations of our study relate to restricting our analyses to individuals of white-European descent to minimize potential bias from population stratification. Therefore, generalizability to other ethnic groups may be limited as a result. While the UK Biobank’s response rate of 5% may not be representative of the general UK population [70], previous studies using this cohort have replicated expected exposure-disease associations [70]. The inclusion of self-reported data in our study is another limitation, as such data may be affected by bias and introduce residual confounding [71]. The use of the MR approach is less affected by confounding and reverse causality than other types of observational studies, which strengthens the validity of our findings [72]. However, it is important to note that the MR approach assumes no horizontal pleiotropy [73], which may result in biased estimates. To address this issue, we engaged several pleiotropy-robust approaches and found no evidence for an association between the genetic score and potential confounders—with additionally genetic instruments being restricted to SNPs with independently replicated evidence for an association with 25(OH)D [74]. MR analyses approximate average effects over the life course, and the true associations may be more complex than presented here. As is appropriate for findings derived from observational studies, associations should be interpreted with due consideration.

## 5. Conclusions

Our study suggests that inflammation might modify the vitamin D and fall risk relationship. Both low and high 25(OH)D levels increased fall risk in individuals with chronic inflammation, with supporting evidence for the association between higher 25(OH)D and increased fall risk in those with chronic inflammation provided via MR analysis. Future research should explore whether the increased fall risk, seen following high-dose vitamin D supplementation in clinical trials, relates to excessive production of VDR-RXR heterodimers that lead to functional retinol deficiency, potentially affecting anti-inflammatory and motor function pathways.

## Figures and Tables

**Table 1:** Demographic characteristics of UK Biobank participants.

		CRP	25(OH)D	CRP ≥5 mg/L	CRP ≥20 mg/L	Falls All	Falls w/CRP ≥5 mg/L	Falls w/CRP ≥20 mg/L
		N = 306,255	N = 307,082	N = 34,961	N = 3519	N = 59,825	N = 8992	N = 940
	N(%)	Geometric Mean (SD)	Mean (SD)	%	%	%	%	%
**All**	307,082 (100)	1.38 (2.89)	49.8 (21.00)	11.42	1.15	19.48	03.51	0.38
**Sex**								
Males	144,418 (47.0)	1.33 (2.79)	49.9 (21.0)	09.85	1.15 *	15.60	02.40	0.30
Females	162,664 (53.0)	1.42 (2.98)	49.8 (20.9)	12.81	1.15	22.93	04.57	0.46
**Age**								
<60	169,475 (55.2)	1.25 (2.93)	48.4 (21.1)	10.41	0.98	17.68	03.01	0.31
≥60	137,607 (44.8)	1.55 (2.80)	51.6 (20.6)	12.66	1.36	21.70	04.14	0.46
**BMI**								
Low 25% 12.1–24.1	76,525 (24.9)	0.79 (2.80)	53.0 (22.0)	05.24	0.86	17.13	01.33	0.24
Mid 50% 24.1–29.8	153,052 (49.8)	1.33 (2.63)	50.9 (20.7)	08.70	0.99	18.17	02.33	0.26
High 25% 29.8–74.7	76,557 (24.9)	2.54 (2.56)	44.4 (19.2)	22.87	1.73	24.18	07.99	0.75
Missing	948 (0.3)	2.25 (3.17)	42.6 (21.3)	23.22	3.39	42.62	16.95	3.20
**Location**								
South ≤ 51° Lat	102,201 (33.3)	1.31 (2.90)	51.4 (20.5)	10.80	1.07	18.79	03.08	0.32
Mid 52–53° Lat	144,370 (47.0)	1.41 (2.87)	49.9 (20.9)	11.54	1.18	20.02	03.69	0.41
North ≥ 54° Lat	60,511 (19.7)	1.43 (2.90)	46.8 (21.6)	12.16	1.20	19.35	03.81	0.41
**Smoking**								
Non-smokers	167,471 (54.5)	1.26 (2.86)	50.0 (20.6)	09.96	0.98	18.63	02.92	0.29
Ex-smokers	108,973 (35.2)	1.46 (2.87)	50.8 (21.0)	12.08	1.30	19.86	03.78	0.44
Current smokers	30,615 (10.0)	1.79 (2.95)	45.2 (21.8)	16.88	1.53	22.58	05.73	0.66
Missing	1023 (0.3)	1.74 (2.88)	50.1 (21.8)	16.21	2.06	25.90	06.54	1.30
**Alcohol**								
Daily	65,476 (21.3)	1.26 (2.82)	51.2 (22.0)	09.57	1.07	18.38	02.60	0.29
1 to 4 times wk	155,379 (50.6)	1.30 (2.84)	50.9 (20.8)	10.11	0.99	17.95	02.80	0.30
1 to 3 times mo	34,047 (11.1)	1.51 (2.90)	48.1 (20.3)	13.07	1.21	20.54	04.09	0.41
Special occasion	32,096 (10.5)	1.74 (2.99)	46.2 (20.4)	16.53	1.63	24.02	06.07	0.66
Never	19,895 (6.5)	1.69 (3.06)	46.0 (21.0)	16.57	1.76	25.83	07.25	0.87
Missing	189 (0.07)	1.79 (2.96)	45.0 (20.5)	16.40	1.59	34.92	08.89	0.00
**Physical activity**								
Low	91,779 (29.9)	1.61 (2.92)	46.3 (20.2)	14.55	1.48	20.14	04.62	0.53
Moderate	149,059 (48.5)	1.28 (2.85)	50.6 (20.8)	09.89	1.00	18.34	02.81	0.29
High	59,492 (19.4)	1.23 (2.80)	54.0 (21.4)	09.02	0.82	19.69	02.63	0.22
Missing	6752 (2.2)	2.23 (3.10)	43.3 (21.3)	23.61	2.84	33.84	12.27	1.76
**Education**								
None	52,009 (17.0)	1.84 (2.83)	50.4 (21.4)	16.29	1.66	23.79	06.02	0.73
NVQ/CSE/A-Levels	108,928 (35.5)	1.43 (2.87)	50.4 (21.2)	11.96	1.14	19.22	03.50	0.34
Degree/professional	143,627 (46.7)	1.20 (2.86)	49.0 (21.0)	09.19	0.96	18.08	02.62	0.29
Missing	2518 (0.82)	1.65 (2.89)	50.4 (21.0)	14.22	1.31	21.84	04.65	0.35
**Townsend index**								
Q1 ↓ Deprivation	76,666 (25.0)	1.27 (2.81)	51.9 (20.7)	09.53	0.93	17.10	02.45	0.22
Q2	76,690 (25.0)	1.32 (2.83)	51.5 (20.7)	10.23	1.02	18.07	02.72	0.30
Q3	76,683 (25.0)	1.36 (2.88)	49.9 (20.8)	11.17	1.11	19.47	03.47	0.37
Q4 ↑ Deprivation	76,682 (25.0)	1.57 (3.01)	46.0 (21.1)	14.73	1.53	23.28	05.47	0.64
Missing	361 (0.1)	1.50 (3.12)	50.0 (20.6)	14.40	2.22	22.16	05.07	0.35

CRP, C-reactive protein; NVQ, National Vocational Qualification; CSE, Certificate of Secondary Education; A-levels, Advanced levels; Q, quartiles, ↑↓ denotes highest and lowest deprivation, respectively. All models were adjusted for sex, age, assessment center, and nuisance factors which could affect serum 25(OH)D measurements, including the month in which the blood sample was taken, fasting time before the blood sample was taken, and sample aliquots; *p* values for all models are ≤0.05, except * *p* = 0.37.

**Table 2:** Phenotypic analyses for the association between log-transformed CRP and categorical 25(OH)D, and the association between 25(OH)D and log-transformed CRP, in the UK Biobank.

25(OH)D nmol/L	N	CRP Geometric Mean (SD). 95% CIs	Simple Model Beta: 95% CIs	Adjusted Model Beta: 95% CIs
<25 nmol/L	35,917	1.63 (3.04). 1.61, 1.65	0.27:0.26, 0.28	0.03:0.02, 0.04
25–49.9 nmol/L	126,950	1.44 (2.88). 1.43, 1.45	0.13:0.12, 0.14	0.005:−0.003, 0.01
50–4.99 nmol/L	107,450	1.29 (2.82). 1.29, 1.3	Reference	Reference
75–99.9 nmol/L	31,843	1.20 (2.90). 1.19, 1.21	−0.08:−0.09, −0.07	0.007:−0.005, 0.02
100–124.9 nmol/L	4324	1.23 (2.95). 1.19, 1.27	−0.05:−0.09, −0.02	0.08:0.05, 0.10
≥125 nmol/L	598	1.31 (3.17). 1.19, 1.44	0.05:−0.04, 0.13	0.18:0.10, 0.26

Fully adjusted non-linearity *p*-value: quadratic non-linearity < 0.001, log ratio test < 0.001. CRP = C-reactive protein. SD = standard deviation. CI = confidence intervals. Simple models were adjusted for sex, age, assessment center, and nuisance factors that could affect 25(OH)D serum measurements, including the month in which the blood sample was taken, fasting time before the blood sample was taken, and sample aliquots for measurement; fully adjusted models were additionally adjusted for educational status, Townsend deprivation index, body mass index, physical activity, alcohol, and smoking.

**Table 3:** Phenotypic analyses for the association of categorical measured 25(OH)D and falls in the UK Biobank, with all falls (adjusted and simple models) and falls concurring with thresholds of C-reactive protein (adjusted and simple models).

Fall Type	10–24.9 nmol/L OR: 95% CI. *p*	25–49.9 nmol/L OR: 95% CI. *p*	Reference	75–99.99 nmol/L OR: 95% CI. *p*	≥100 nmol/L OR: 95% CI. *p*
**All falls** ***Adjusted***	1.18:1.14, 1.22. <0.001	1.05:1.03, 1.08. <0.001	*Ref.*	1.00:0.97, 1.04. 0.83	1.00:0.93, 1.09. 0.92
**All falls** ***Simple***	1.32:1.28, 1.37. <0.001	1.10:1.08, 1.13. <0.001	*Ref.*	0.97:0.94, 1.01. 0.12	0.97:0.90, 1.05. 0.40
**With CRP** ***Adjusted***					
≥05 mg/L	1.25:1.15, 1.34. <0.001	1.07:1.01, 1.13. 0.02	*Ref.*	1.04:0.95, 1.14. 0.40	1.31:1.07, 1.60. 0.009
≥10 mg/L	1.33:1.17, 1.50. <0.001	1.04:0.95, 1.14. 0.39	*Ref.*	1.17:1.01, 1.36. 0.03	1.75:1.30, 2.35. <0.001
≥15 mg/L	1.39:1.18, 1.65. <0.001	1.05:0.93, 1.20. 0.42	*Ref.*	1.30:1.07, 1.60. 0.01	1.93:1.30, 2.87. 0.001
≥20 mg/L	1.29:1.03, 1.61. 0.03	1.01:0.85, 1.20. 0.91	*Ref.*	1.53:1.19, 1.96. 0.001	2.40:1.50, 3.86. <0.001
**With CRP** ***Simple***					
≥05 mg/L	2.28:2.12, 2.45. <0.001	1.43:1.35, 1.51. <0.001	*Ref.*	0.86:0.79, 0.94. 0.001	1.00:0.82, 1.22. 0.97
≥10 mg/L	2.55:2.27, 2.86. <0.001	1.41:1.29, 1.55. <0.001	*Ref.*	0.96:0.83, 1.11. 0.63	1.34:1.00, 1.79. 0.05
≥15 mg/L	2.64:2.25, 3.10. <0.001	1.41:1.24, 1.61. <0.001	*Ref.*	1.08:0.89, 1.32. 0.45	1.50:1.01, 2.22. 0.04
≥20 mg/L	2.45:1.98, 3.02. <0.001	1.35:1.14, 1.60. 0.001	*Ref.*	1.28:1.00, 1.65. 0.05	1.89:1.18, 3.02. 0.008

CRP = C-reactive protein. OR = odds ratio. CI = confidence intervals. Simple models were adjusted for sex, age, assessment center, and nuisance factors that could affect 25(OH)D serum measurements, including the month in which the blood sample was taken, fasting time before the blood sample was taken, and sample aliquots for measurement; fully adjusted models were additionally adjusted for educational status, Townsend deprivation index, body mass index, physical activity, alcohol, and smoking.

## Data Availability

This research has been conducted using the UK Biobank resource under application number 20175. All data and code will be available to approved users upon application to the UK Biobank.

## Supplementary Materials

The following supporting information can be downloaded at: https://www.mdpi.com/article/10.3390/nu17010038/s1, Figure S1: Study population and participant inclusion criteria; Figure S2: Latitudinal zoning of the UK Biobank Assessment Centers; Figure S3: Selection of variants for the genetic instrument for measured 25(OH)D concentrations; Figure S4: Mendelian randomization assumption diagram; Figure S5: Distribution of the vitamin D genetic score (mapped on an X-axis by increasing number of alleles) in the UK Biobank (A), and the association of the vitamin D genetic score (in six groups—lowest to highest number of alleles) with measured 25(OH)D concentrations in the UK Biobank (B); Table S1: Genome-wide significant vitamin D variants used for the genetic instruments for measured 25(OH)D concentrations; Table S2: Association of the vitamin D genetic score with potential confounders in the UK Biobank; Table S3: Mendelian randomization analysis for the association of genetically predicted 25(OH)D with falls concurring with C-reactive protein > 20 mg/L in the UK Biobank-122 SNP genetic score and leave block out analysis; Table S4: Functional blocks used in the leave-block-out analyses; Table S5: Phenotypic analyses showing the prevalence of falls across CRP ranges, and the association between falls and categorical CRP, in the UK Biobank; Table S6: Fully adjusted association between continuous 25(OH)D and falls, stratified above and below CRP thresholds; Table S7: Fully adjusted association between continuous 25(OH)D and falls, with the outcome of falls concurring with inflammation across increasing CRP thresholds; Table S8: Phenotypic sensitivity analyses for the association of categorical 25(OH)D and falls in the UK Biobank, with the outcome of falls concurring to those with rheumatoid arthritis (adjusted and simple models); Table S9: Phenotypic analyses for the association of categorical measured 25(OH)D and falls in the UK Biobank, with falls concurring with CRP < 5 mg/L.
