# Supplementary material for: Vitamin D, C-Reactive Protein, and Increased Fall Risk: A Genetic Epidemiological Study
Source: Nutrients. 2024 Dec 26;17(1):38. doi: 10.3390/nu17010038 (PMC11722653; doi:10.3390/nu17010038)
Supplement: Supplementary file 1 [file nutrients-17-00038-s001.zip › nutrients-3338768-supplementary.pdf]

Supplementary content for: Vitamin D, C-Reactive Protein, and Increased Fall Risk: A Genetic Epidemiological Study:

Supplementary Methods: Measurement of vitamin D status	**03**
Supplementary Methods: Covariates	**03**
Supplementary Methods: Genetic instruments using 35 variants	**04**
Supplementary Methods: Genetic instrument using 122 vitamin D GWAS variants	**05**
Supplementary Methods: Linear Mendelian randomization	**05**
Supplementary Methods: Leave-block-out analysis	**08**
**Figure S1.** Study population and participant inclusion criteria.	**10**
**Figure S2.** Latitudinal zoning of the UK Biobank Assessment Centers.	**11**
**Figure S3.** Selection of variants for the genetic instrument for measured 25(OH)D concentrations.	**12**
**Figure S4.** Mendelian randomization assumption diagram.	**13**
**Figure S5.** Distribution of the vitamin D genetic score (mapped on an X-axis by increasing number of alleles) in the UK Biobank (A), and the association of the vitamin D genetic score (in six groups – lowest to highest number of alleles) with measured 25(OH)D concentrations in the UK Biobank (B).	**14**
**Table S1.** Genome-wide significant vitamin D variants used for the genetic instruments for measured 25(OH)D concentrations.	**15**
**Table S2.** Association of the vitamin D genetic score with potential confounders in the UK Biobank.	**19**
**Table S3**. Mendelian randomization analysis for the association of genetically predicted 25(OH)D with falls concurring with C-reactive protein > 20 mg/L in the UK Biobank - 122 SNP genetic score and leave block out analysis.	**20**
**Table S4.** Functional blocks used in the leave-block-out analyses.	**21**
**Table S5.** Phenotypic analyses showing the prevalence of falls across CRP ranges, and the association between falls and categorical CRP, in the UK Biobank.	**22**
**Table S6.** Fully adjusted association between continuous 25(OH)D and falls, stratified above and below CRP thresholds.	**22**
**Table S7.** Fully adjusted association between continuous 25(OH)D and falls, with the outcome of falls concurring with inflammation across increasing CRP thresholds.	**23**
**Table S8.** Phenotypic sensitivity analyses for the association of categorical 25(OH)D and falls in the UK Biobank, with the outcome of falls concurring to those with rheumatoid arthritis (adjusted and simple models).	**24**
**Table S9.** Phenotypic analyses for the association of categorical measured 25(OH)D and falls in the UK Biobank, with falls concurring with CRP <5mg/L.	**24**
**References**	**25**

Authors:

Joshua P. Sutherland [1], Ang Zhou [1,2,3] and Elina Hyppönen [1,2,*]

[1] Australian Centre for Precision Health, Unit of Clinical and Health Sciences, University of South Australia, Adelaide, SA 5000, Australia
[2] South Australian Health and Medical Research Institute, Adelaide, SA 5000, Australia
[3] Medical Research Council Biostatistics Unit, University of Cambridge, Cambridge CB2 0SR, UK
[*] Correspondence: elina.hypponen@unisa.edu.au; Tel.: +61-8-830-22518

Supplementary Methods:

Measurement of vitamin D status

The LIAISON XL 25(OH)D assay (DiaSorin, Stillwater, USA) [1] was used to measure baseline serum 25(OH)D concentrations in blood samples. This fully automated, chemiluminescence immunoassay device utilizes magnetic microparticle separation technology.

- The blood samples used had a mean fasting time of 3.8 hours (SD 2.4).
- Assay precision was evaluated using the CLSI EP5-A2 protocol, with 6 samples and 2 controls. The intra-run coefficient of variation (CV) was 2.3%, and the total CV was 7.8%.
- The functional sensitivity of the LIAISON XL assay is ≤ 4.0 ng/ml, in accordance with Clinical Laboratory and Standards Institute (CLSI) EP17-A protocols. [1]

Covariates

The covariates used in adjustments for this study were collected at baseline, with many derived from self-reported touchscreen questionnaires (age, sex, physical activity, smoking, alcohol), residential address data (Townsend deprivation index), [2] and physical assessments (height and weight). The assessment centre at which baseline assessment occurred was itself used as a covariate (with the centers forming categories, as outlined in **Figure S2**). Height data was obtained using a Seca 202 stadiometer, and weight was acquired using the body composition analyser Tanita BC-418MA, or via body weight scales in instances where body composition analysis was not used. BMI was calculated as body weight [in kilograms] divided by height

squared [in meters]) [3] and divided into quartiles (with the two middle quartiles combined into a single 'mid' BMI category)

For Townsend deprivation index, each participant was assigned a score corresponding to socioeconomic data attributed to their postcode; this was derived from the preceding UK census of population and housing. [2, 4] Longitudinal location was determined based on latitudinal zoning of the assessment center (Figure S2). The genetic covariates, and 25(OH)D concentrations were analyzed from samples collected at baseline.

<u>Genetic instruments using 35 variants</u>

Our genetic score for measured 25(OH)D serum concentration was constructed using a weighted approach that involved collating common autosomal SNPs (minor allele frequency $\geq$ 5%) from a GWAS analysis conducted on the UK Biobank dataset, wherein 134 indpendent loci were identified. [5] To ensure the robustness of our findings, we replicated SNP analyses in the previous SUNLIGHT consortium GWAS, [6] and this left us with 35 SNPs that showed consistent direction and a p value of < 0.05 (**Figure S3**).

- So as to avoid bias due to sample overlap, we utilized the SUNLIGHT consortium meta-analyses derived effect estimates for the SNP association with measured 25(OH)D, as the individual SNP weights. [7]
- This enabled us to ensure that the observed GWAS signals were robust.
- We computed the independently derived weighted average of the number of measured 25(OH)D-increasing alleles for an individual, and then multiplied this by the number of available variants.

Genetic instrument using 122 vitamin D GWAS variants

For sensitivity analyses, we developed an alternative genetic instrument by including a broader set of variants that are associated with measured 25(OH)D concentrations. This new instrument comprises of 122 autosomal single nucleotide polymorphisms (SNPs) that were identified from the UK Biobank dataset [5] (Figure S3).

- To avoid any potential bias arising from using internal weights, we used a 10-fold cross-validation approach [7] to construct the score.
- This process involved randomly dividing the samples into 10 equal sub-samples and using the weights from the remaining 9 sub-samples to calculate the scores.
- Information regarding the 122 SNPs used in this instrument is available in **Table S1**.

Mendelian randomization:

In our study we employed a combination of a Genetic Score One Sample method and SNP-based two-sample Mendelian randomization (MR) approaches.

For the Genetic Score One Sample method, we utilized the ratio of coefficients method in computing the MR estimate, [8] where we calculated the vitamin D genetic score – measured 25(OH)D and the vitamin D genetic score – outcome association estimates and then used these to compute the ratio estimator.

Vitamin D genetic score – measured 25(OH)D and the vitamin D genetic score – outcome association estimates were computed by regressing 25(OH)D and outcome on the vitamin D genetic score, respectively. In these regression models, we adjusted for various factors that could affect measured 25(OH)D, including age, sex, assessment centre, SNP array, top 40 genetic principal components, and nuisance factors like the month in which the blood sample was taken, fasting time before blood sample was taken, and sample aliquots for measurement.

To address potential horizontal pleiotropy, we also implemented five SNP-based two-sample MR methods, including the inverse-variance weighted method, Mendelian randomization Egger method, weighted median Mendelian randomization method, weighted mode method, and Mendelian randomization pleiotropy residual sum and outlier (MR Presso) test. These methods are complementary to each other and are robust to different patterns of horizontal pleiotropy. Consistency amongst the methods is indicative of robust evidence of causal association. It is important to note that in our analysis, both SNP-25(OH)D and the SNP-falls association were derived from the UK Biobank. To avoid potential bias due to overlapping samples [9], we computed SNP-Fall association estimates in the full sample but took SNP-25(OH)D association estimates from controls only [9].

In contrast to the above outlined Genetic Score One Sample method analysis the following is an overview of the two sample MR approaches:

- SNP-level information was used, and SNP-exposure and SNP-outcome association estimates were taken as inputs.

- The two-sample MR analyses used SNP-25(OH)D and SNP-outcome association estimates from UK Biobank.
- SNP-Fall association estimates were computed in the full sample, but SNP-25(OH)D association estimates were taken from controls only.
- All five two-sample MR methods were conducted using the 35 vitamin D variants used to construct the vitamin D genetic score (Table S1).

Here, we provide a brief overview of each of the employed two-sample MR approaches:

1. Inverse variance-weighted (IVW) method:
    - Assumes that there is no pleiotropy of variants or that the net pleiotropy is zero.
    - The estimator is obtained by the weighted regression of the effect estimates of variant-outcome association on the effect estimates of variant-exposure association, with the weight being the inverse variance of the variant's ratio estimate. [10]
2. Mendelian randomization Egger method:
    - Similar to the IVW method, the Mendelian randomization Egger method assumes that the regression model includes an intercept term, which represents the average pleiotropic effect. [11]
    - This method can account, however, for directional pleiotropy, which introduces additional untestable assumptions stating that the pleiotropic effect must be independent of the variant-exposure association.

3. Mendelian randomization Presso method:
    - Mendelian randomization Presso [12] applies an iterative version of the IVW method, where each variant is omitted from the analysis to assess its contribution to heterogeneity.
    - The residual sum of squares (RSS) is calculated at each iteration and used as a measure of heterogeneity.
    - Variants that produce substantially large RSS values are considered outliers and removed from the analysis.
    - The causal estimate in MR Presso is obtained by applying the IVW method with all outlying variants removed.
4. Weighted mode method:
    - Variants are clustered into groups by the similarity of their ratio estimates. [13]
    - Variants are weighted by the inverse variance of their ratio estimates.
    - Clustering is preformed using a normal kernel density function, with its spread depending on a band with parameter.
    - The causal effect is estimated based on the cluster with the largest weight.
    - An unbiased causal effect will be returned if the variants within the cluster with the largest weight are valid instruments.
5. Weighted median-based method:
    - Variants are ranked by their ratio estimates, with the causal estimate being the median of the distribution. [14]

- To ensure a consistent estimate from the weighted median method, it is necessary that valid instruments contribute to at least 50% of the total weight.
- Median is taken from a distribution of the ration estimates in which variants with more precise ratio estimates receive more weight.

Leave-block-out analysis

In our leave-block-out analysis, we first grouped variants into functional blocks based on their associated traits, as identified by the PhenoScanner V2 database. [15] To explore sensitivity of the analysis, we then systematically removed one block of variants at a time and repeated the non-linear Mendelian randomization analysis using the vitamin D genetic score constructed from the remaining, non-removed variants. We repeated this process for all functional blocks.

PhenoScanner V2 is a publicly available database that curates results from large-scale genetic association studies. [15] On 09/09/2021, we searched for evidence of variant-disease/trait associations for all 35 variants in this database. Using a GWAS threshold of $P < 5E-08$, we found that 20 variants were associated with traits other than serum 25(OH)D (**Table S3**). Based on the associated traits, we grouped the variants into four functional blocks: blood, lipids/metabolic, renal traits, and 'unclassified' (with the latter group being for variants whose associated traits did not fall into one of these 3 blocks) (**Table S4**).

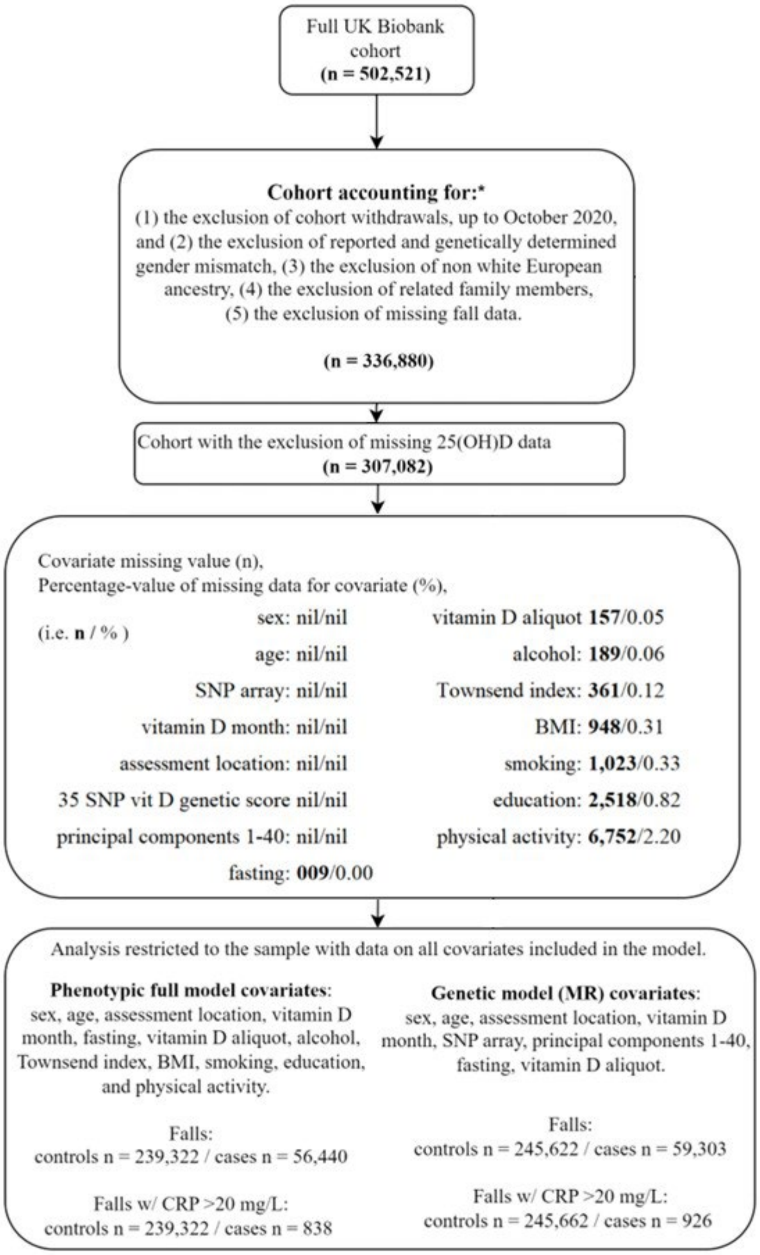

Figure S1. Study population and participant inclusion criteria.

*Exclusion was done in a sequential order. MR = Mendelian randomization.

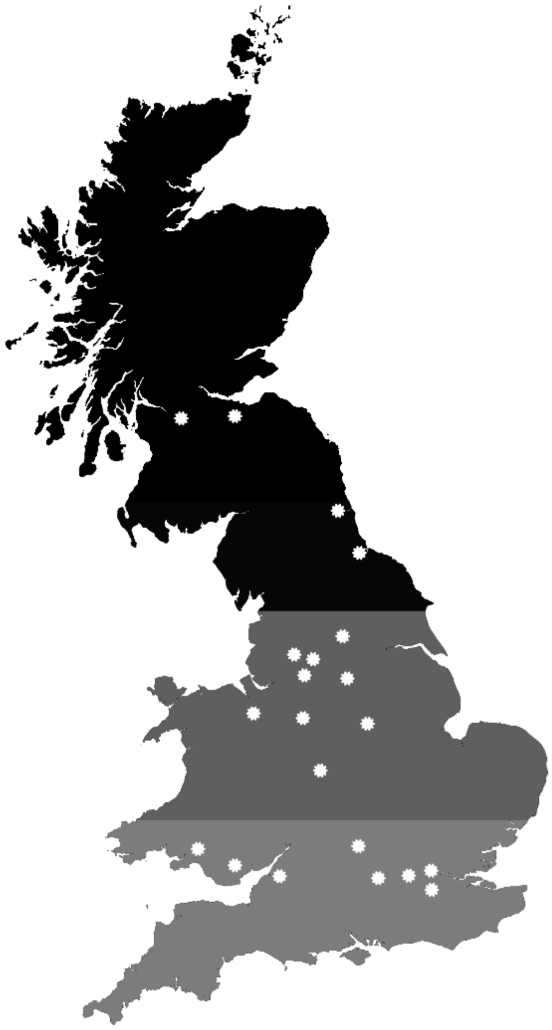

Figure S2. Latitudinal zoning of the UK Biobank Assessment Centers.

Categorical latitudinal zoning, in ascending order of light to dark: ≤ 51° / 52°-53° / 54°-≥ 55°.
≤ 51° (Croydon, Barts, Hounslow, Reading, Oxford, Bristol, Cardiff, Swansea), 52°-53° (Birmingham, Stoke, Nottingham, Wrexham, Liverpool, Bury, Manchester, Sheffield, Leeds, Stockport), and 54°-≥ 55° (Middlesborough, Newcastle, Glasgow, Edinburgh).

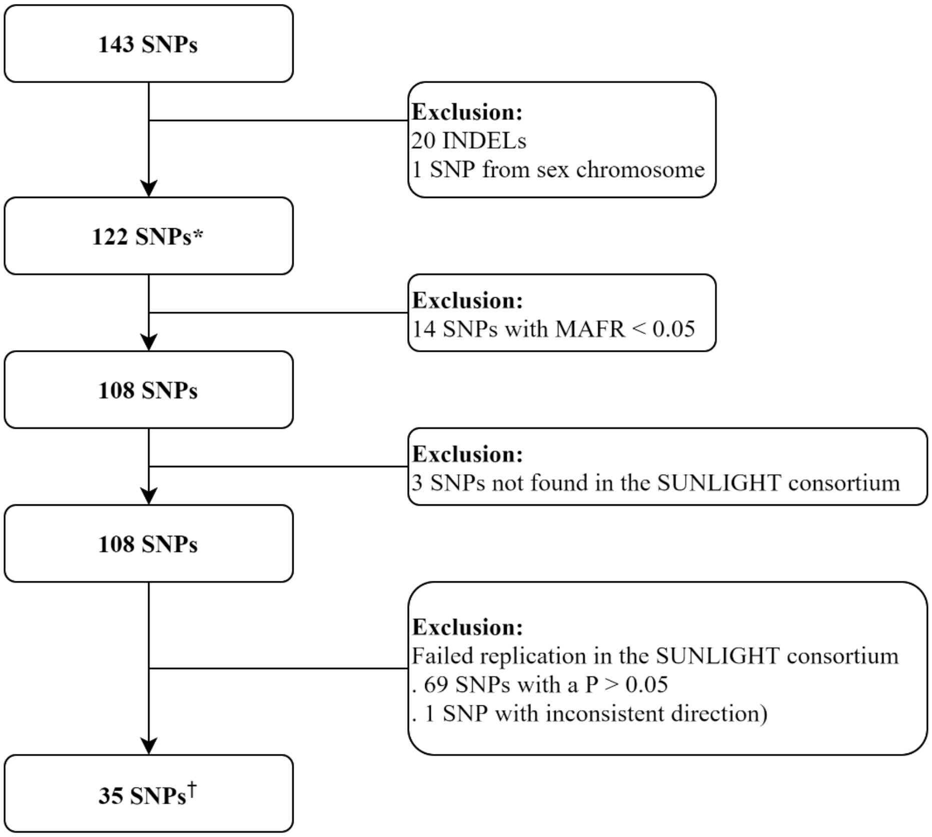

Figure S3. Selection of variants for the genetic instrument for measured 25(OH)D concentrations.

SNP: single nucleotide polymorphism. *SNPs used for the vitamin D genetic score in the primary analysis; †SNPs used for the 122 SNP version of the vitamin D genetic score in the sensitivity analysis. INDEL: insertion and deletion; MAF: minor allele frequency.

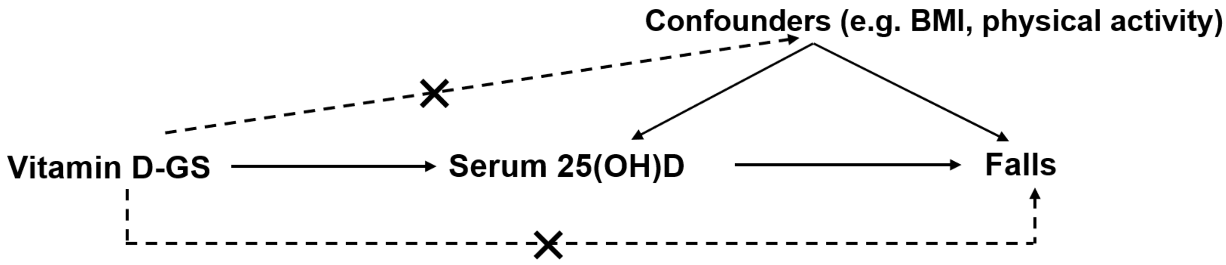

Figure S4. Mendelian randomization assumption diagram.

Valid causal inference from the Mendelian randomization analysis relies on 3 key assumptions: [16] 1) vitamin D genetic score associates with measured 25(OH)D concentrations; 2) vitamin D genetic score has no direct effect on outcome; 3) vitamin D genetic score does not associate with confounders of measured 25(OH)D and outcome. Vitamin D-GS: vitamin D genetic score.

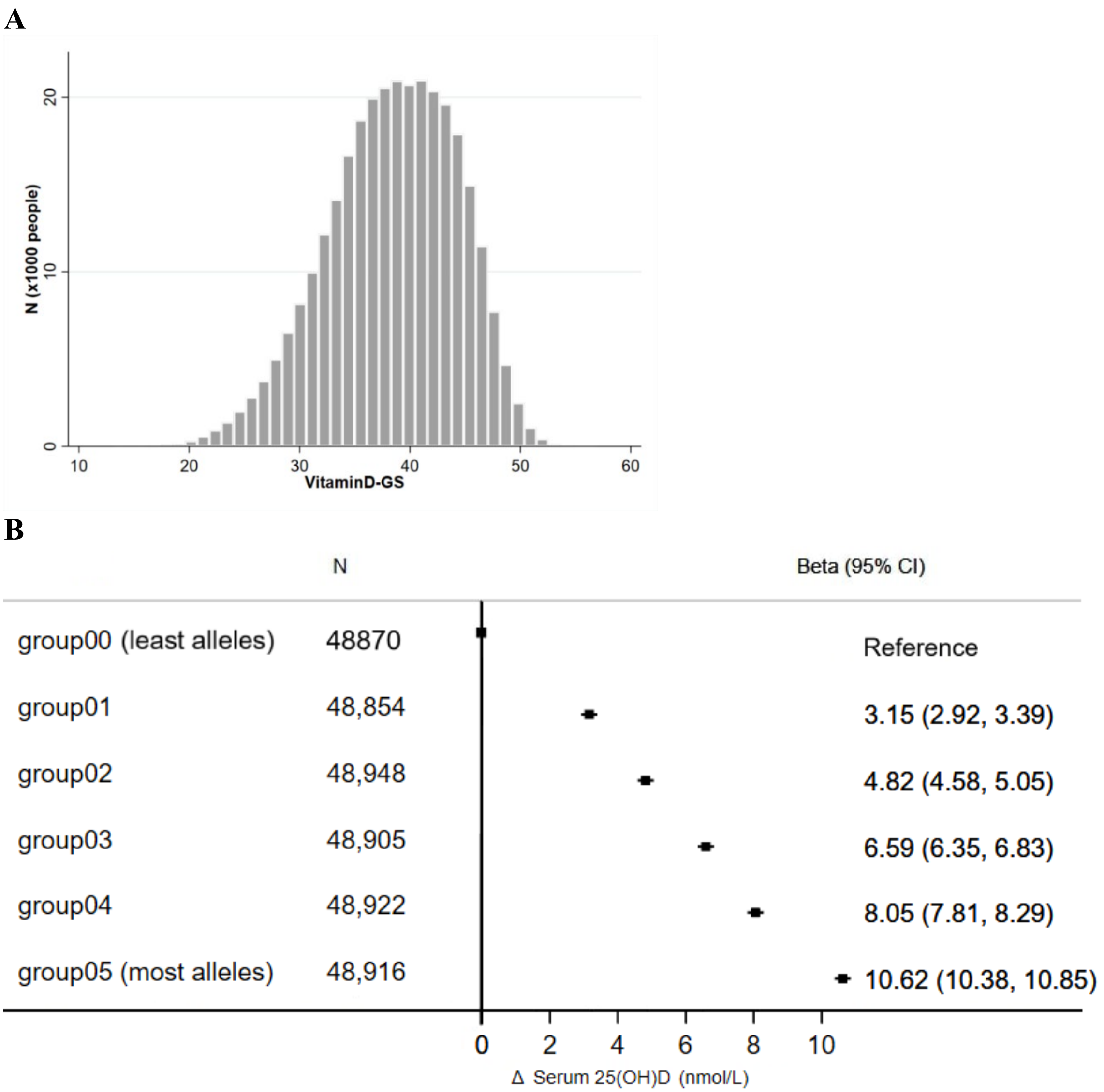

Figure S5. Distribution of the vitamin D genetic score (mapped on an X-axis by increasing number of alleles) in the UK Biobank (A), and the association of the vitamin D genetic score (in six groups – lowest to highest number of alleles) with measured 25(OH)D concentrations in the UK Biobank (B).

The association was tested using the linear regression, with the model adjusted for age, sex, genotyping array, assessment center location, top 40 genetic principal components, birth location, and nuisance factors which could affect serum 25(OH)D measurements, including month in which blood sample was taken, fasting time before blood sample was taken, and sample aliquots for measurement. Error bars are 95% confidence intervals. Vitamin D-GS: Vitamin D Genetic Score.

Table S1. Genome-wide significant vitamin D variants used for the genetic instruments for measured 25(OH)D concentrations.

No	SNP	CHR	BP	Gene	A1	A2	A1F	UK Biobank*			SUNLIGHT Consortium†			SNPs for 35 SNP genetic score	SNPs for 122 SNP genetic score
								Beta	SE	P	SNPs for 35 SNP score	SE	P		
1	rs6671730[a]	1	2339139	*PEX10*	G	A	0.565714	0.0147881	0.00201077	1.92E-13	0.0061	0.0023	0.006652	Yes	Yes
2	rs35408430	1	17560195	*PADI1*	C	T	0.657806	0.0214952	0.00209979	1.36E-24	0.0236985	0.00564768	0.00002715	Yes	Yes
3	rs7522116	1	41835685	*FOXO6*	C	T	0.433767	0.0134641	0.00202533	2.97E-11	0.0116727	0.00540416	0.03077654	Yes	Yes
4	rs7528419	1	109817192	*CELSR2*	G	A	0.224671	0.0197401	0.00238729	1.35E-16	0.0179046	0.00644566	0.0054732	Yes	Yes
5	rs1933064	1	152301576	*FLG-AS1*	A	G	0.46961	0.015731	0.00203195	9.80E-15	0.0155068	0.00539368	0.00404027	Yes	Yes
6	rs76798800	1	154994978	*DCST2*	G	T	0.733745	0.0121989	0.00225962	6.71E-08	0.0173898	0.00617041	0.00482841	Yes	Yes
7	rs6672758	1	230303512	*GALNT2*	T	C	0.800872	0.0175857	0.00250898	2.40E-12	0.0156301	0.00666121	0.01895423	Yes	Yes
8	rs727857	2	58981967	*LINC01122*	G	A	0.388511	0.0140184	0.00206152	1.05E-11	0.0109131	0.00550772	0.04754487	Yes	Yes
9	rs1047891	2	211540507	*CPS1*	C	A	0.684179	0.0152142	0.00214041	1.18E-12	0.0126572	0.00572581	0.02706743	Yes	Yes
10	rs2012736	2	234622379	*UGT1A5, UGT1A6, UGT1A7, UGT1A8, UGT1A9, UGT1A10*	C	A	0.919186	0.0483073	0.00366555	1.16E-39	0.0384413	0.01038238	0.00021344	Yes	Yes
11	rs6782190	3	85639672	*CADM2*	G	A	0.352488	0.0172156	0.00208415	1.45E-16	0.0206718	0.00562451	0.00023756	Yes	Yes
12	rs705117	4	72608115	*GC*	C	T	0.1477	0.0334179	0.00280601	1.06E-32	0.0269429	0.00744137	0.00029382	Yes	Yes
13	rs1352846	4	72617775	*GC*	A	G	0.708567	0.193471	0.00219074	0	0.2221843	0.00589771	1.40E-310	Yes	Yes
14	rs78151190	6	25619007	*CARMIL1 (LRRC16A)*	A	C	0.871284	0.0168754	0.00297406	1.39E-08	0.0187117	0.00829773	0.02413132	Yes	Yes
15	rs75741381	7	100809458	*VGF*	C	G	0.852362	0.0166065	0.00282521	4.15E-09	0.0214474	0.00736197	0.00357669	Yes	Yes
16	rs12056768	8	116988527	*LINC00536*	T	G	0.417091	0.0234029	0.00202418	6.44E-31	0.0176616	0.00545433	0.00120331	Yes	Yes
17	rs77532868	10	88081438	*GRID1*	T	C	0.054042	0.0265692	0.00440069	1.57E-09	0.0280553	0.01353113	0.03813628	Yes	Yes
18	rs12794714	11	14913575	*CYP2R1*	G	A	0.578197	0.0878964	0.00201629	0	0.0702488	0.00540376	1.22E-38	Yes	Yes
19	rs61891388	11	66079818	*RP11-867G23.13*	G	T	0.455921	0.0125532	0.00200799	4.06E-10	0.0114254	0.00538961	0.03401532	Yes	Yes
20	rs1660839	11	71094232	*AP002387.1*	A	G	0.248849	0.0292665	0.00230557	6.40E-37	0.014173	0.00623639	0.02304867	Yes	Yes
21	rs12803256	11	71132868	*AP002387.1*	G	A	0.776732	0.104243	0.00239998	0	0.0839119	0.00602549	4.39E-44	Yes	Yes
22	rs12798050[b]	11	71223256	*S100A11P3*	T	C	0.830503	0.109998	0.00264849	0	0.0348	0.0024	1.00E-47	Yes	Yes
23	rs72997623	11	75488054	*DGAT2*	A	C	0.084662	0.0276158	0.00358139	1.25E-14	0.0200157	0.00937765	0.03280964	Yes	Yes
24	rs1149605	11	76485216	*RP11-21L23.4*	C	T	0.170397	0.0220166	0.00266133	1.31E-16	0.0209786	0.0072426	0.00377288	Yes	Yes
25	rs10859995	12	96375682	*HAL*	T	C	0.417366	0.0403465	0.0020206	1.05E-88	0.036551	0.00540543	1.36E-11	Yes	Yes
26	rs8018720	14	39556185	*SEC23A*	G	C	0.176673	0.0378247	0.00260904	1.26E-47	0.040852	0.00705183	6.91E-09	Yes	Yes
27	rs261291	15	58680178	*ALDH1A2*	T	C	0.644772	0.0273653	0.00208561	2.50E-39	0.0113468	0.0056366	0.04410853	Yes	Yes
28	rs77924615	16	20392332	*PDILT*	G	A	0.806515	0.0166321	0.00255158	7.11E-11	0.0195535	0.00670691	0.00355194	Yes	Yes
29	rs212100	19	48376995	*SULT2A1*	T	C	0.164001	0.0661522	0.00269018	1.61E-133	0.0193875	0.00719712	0.00706453	Yes	Yes
30	rs10426	19	51517798	*KLK10*	A	G	0.213433	0.0256629	0.00243056	4.64E-26	0.0146449	0.0065379	0.02509092	Yes	Yes
31	rs6123359	20	52714706	*BCAS1*	G	A	0.102225	0.0341831	0.00331429	6.10E-25	0.0373636	0.00940288	0.00007078	Yes	Yes

No	SNP	CHR	BP	Gene	A1	A2	A1F	UK Biobank*			SUNLIGHT Consortium[†]			SNPs for 35 SNP genetic score	SNPs for 122 SNP genetic score
								Beta	SE	P	SNPs for 35 SNP score	SE	P		
32	rs17216707	20	52732362	*CYP24A1*	T	C	0.817316	0.0376264	0.00263713	3.47E-46	0.0646902	0.0066412	2.02E-22	Yes	Yes
33	rs2585442	20	52737123	*CYP24A1*	G	C	0.240654	0.0356675	0.00237687	6.70E-51	0.0381477	0.00635676	1.96E-09	Yes	Yes
34	rs2762943	20	52790786	*CYP24A1*	G	T	0.923071	0.0457231	0.00373798	2.10E-34	0.032534	0.01442103	0.02406994	Yes	Yes
35	rs2074735	22	31535872	*PLA2G3*	C	G	0.064096	0.0278196	0.00407045	8.23E-12	0.0213678	0.01054517	0.04273241	Yes	Yes
36	rs11591147	1	55505647	*PCSK9*	T	G	0.018041	0.0450903	0.00747745	1.64E-09	-	-	-	No	Yes
37	rs2131925	1	63025942	*DOCK7*	G	T	0.356375	0.0229402	0.0020845	3.61E-28	-	-	-	No	Yes
38	rs140371183	1	152098428	*PUDPP2 (HDHD1P2)*	G	A	0.032179	0.0870147	0.00565335	1.86E-53	-	-	-	No	Yes
39	rs12123821	1	152179152	*FLG-AS1, RP11-107M16.2*	T	C	0.047527	0.0785529	0.00467652	2.55E-63	-	-	-	No	Yes
40	rs61816761	1	152285861	*FLG-AS1,FLG*	A	G	0.015926	0.12315	0.00804767	7.35E-53	-	-	-	No	Yes
41	rs10908419	1	154567699	*ADAR*	G	A	0.510067	0.012342	0.00199206	5.81E-10	-	-	-	No	Yes
42	rs11264322	1	155087933	*Y_RNA*	G	A	0.570235	0.0093848	0.0020223	3.47E-06	-	-	-	No	Yes
43	rs10908465	1	155389688	*ASH1L*	T	C	0.267332	0.0168816	0.00224924	6.12E-14	-	-	-	No	Yes
44	rs867772	1	220972343	*MARC1*	A	G	0.315487	0.0146031	0.00215141	1.14E-11	-	-	-	No	Yes
45	rs7604788	2	21190024	*RP11-116D2.1*	T	C	0.033433	0.0336737	0.0055518	1.32E-09	-	-	-	No	Yes
46	rs541041	2	21294975	*APOB*	G	A	0.180761	0.0154585	0.00258635	2.27E-09	-	-	-	No	Yes
47	rs1260326	2	27730940	*GCKR*	C	T	0.606565	0.0206128	0.00203644	4.41E-24	-	-	-	No	Yes
48	rs11127186	2	28881407	*AC074011.2*	C	T	0.495795	0.0109023	0.00203379	8.30E-08	-	-	-	No	Yes
49	rs2710651	2	63166379	*EHBP1*	G	A	0.471877	0.0114568	0.00199623	9.51E-09	-	-	-	No	Yes
50	rs3849374	2	101443397	*NPAS2, AC092168.2*	G	C	0.821971	0.0161021	0.00261564	7.46E-10	-	-	-	No	Yes
51	rs7569755	2	118648261	*HTR5BP*	A	G	0.29058	0.01425	0.00221206	1.18E-10	-	-	-	No	Yes
52	rs13060130	3	84440527	*AC108696.1*	C	T	0.860311	0.0149893	0.00287224	1.80E-07	-	-	-	No	Yes
53	rs9861009	3	141654685	*TFDP2 (RP11-271K21.11)*	C	T	0.727515	0.0140213	0.00225294	4.86E-10	-	-	-	No	Yes
54	rs78649910	4	3482213	*DOK7*	T	A	0.893821	0.0211949	0.00325203	7.15E-11	-	-	-	No	Yes
55	rs4364259	4	15892159	*RP11-442P12.1*	A	G	0.202148	0.0159119	0.00250595	2.16E-10	-	-	-	No	Yes
56	rs4616820	4	57745481	*REST*	C	T	0.535046	0.012286	0.00201755	1.13E-09	-	-	-	No	Yes
57	rs35057908	4	69372082	*UGT2B29P*	T	A	0.431309	0.0110085	0.00202343	5.31E-08	-	-	-	No	Yes
58	rs13104260	4	70348090	*UGT2B4*	A	G	0.256938	0.0072531	0.00228347	0.00149139	-	-	-	No	Yes
59	rs11732896	4	88287993	*HSD17B11*	G	A	0.701209	0.0160047	0.00217341	1.79E-13	-	-	-	No	Yes
60	rs28364331	4	100201295	*RP11-696N14.1, ADH1A*	G	A	0.018086	0.068614	0.00747169	4.19E-20	-	-	-	No	Yes
61	rs1229984	4	100239319	*ADH1B*	T	C	0.024889	0.0450574	0.00637113	1.53E-12	-	-	-	No	Yes
62	rs10070734	5	87940026	*LINC00461*	C	T	0.709531	0.0132137	0.00219753	1.82E-09	-	-	-	No	Yes
63	rs31612	5	108996643	*AC012603.1*	T	C	0.825562	0.014528	0.00264902	4.15E-08	-	-	-	No	Yes
64	rs72834856	6	22801858	*RP1-209A6.1*	T	G	0.927936	0.0249871	0.00385103	8.67E-11	-	-	-	No	Yes

No	SNP	CHR	BP	Gene	A1	A2	A1F	UK Biobank*			SUNLIGHT Consortium†			SNPs for 35 SNP genetic score	SNPs for 122 SNP genetic score
								Beta	SE	P	SNPs for 35 SNP score	SE	P		
65	rs28374650	6	32623367	*HLA-DQB1*	C	T	0.756438	0.0135623	0.00232502	5.44E-09	-	-	-	No	Yes
66	rs9476310	6	57767576	*RP11-325M4.2*	T	C	0.511363	0.0117571	0.00200093	4.21E-09	-	-	-	No	Yes
67	rs9490317	6	121859499	*RNU4-76P*	C	T	0.445894	0.011051	0.00201182	3.95E-08	-	-	-	No	Yes
68	rs2248551	6	131924689	*MED23*	G	A	0.834778	0.0233623	0.0026823	3.04E-18	-	-	-	No	Yes
69	rs10085881	7	21577960	*DNAH11*	T	C	0.717815	0.0145575	0.00223829	7.83E-11	-	-	-	No	Yes
70	rs7784802	7	64015379	*ZNF680*	T	A	0.360991	0.0138131	0.00207209	2.62E-11	-	-	-	No	Yes
71	rs6966728	7	104618318	*LINC01004*	C	T	0.537368	0.0117532	0.00203758	8.01E-09	-	-	-	No	Yes
72	rs2346264	7	133536351	*EXOC4*	A	C	0.217315	0.0138826	0.00243596	1.21E-08	-	-	-	No	Yes
73	rs34290760	8	9185179	*RP11-115J16.1*	C	G	0.970888	0.0334615	0.00593241	1.70E-08	-	-	-	No	Yes
74	rs804281	8	11611865	*GATA4*	G	A	0.583605	0.0132996	0.00202139	4.72E-11	-	-	-	No	Yes
75	rs28692966	8	25892919	*EBF2*	A	G	0.252936	0.0148311	0.00230018	1.14E-10	-	-	-	No	Yes
76	rs2725371	8	30854033	*PURG*	G	A	0.697691	0.0118392	0.00218287	5.84E-08	-	-	-	No	Yes
77	rs4738684	8	59393273	*CYP7A1*	G	A	0.665522	0.0124102	0.00211491	4.41E-09	-	-	-	No	Yes
78	rs13284054	9	107669073	*ABCA1*	C	T	0.117727	0.0175688	0.00313411	2.07E-08	-	-	-	No	Yes
79	rs10887718	10	82042624	*MAT1A*	C	T	0.471787	0.0111219	0.00199828	2.61E-08	-	-	-	No	Yes
80	rs3925446	10	91495322	*KIF20B*	A	G	0.199129	0.0152166	0.00249607	1.09E-09	-	-	-	No	Yes
81	rs4418728	10	94839724	*CYP26A1*	T	G	0.451688	0.0109611	0.00200006	4.24E-08	-	-	-	No	Yes
82	rs61883501	11	13882754	*RP11-98J9.2, RP11-98J9.3*	A	C	0.966768	0.0017709	0.00553993	0.749232	-	-	-	No	Yes
83	rs116970203	11	14876718	*PDE3B*	G	A	0.972844	0.376873	0.00612043	0	-	-	-	No	Yes
84	rs117576073	11	14912573	*CYP2R1*	G	T	0.987265	0.147177	0.00886081	5.91E-62	-	-	-	No	Yes
85	rs78168201	11	70971149	*SHANK2*	T	C	0.013766	0.0893469	0.00864963	5.18E-25	-	-	-	No	Yes
86	rs964184	11	116648917	*ZPR1 (ZNF259)*	C	G	0.868376	0.0431755	0.00294423	1.09E-48	-	-	-	No	Yes
87	rs613808	11	116710968	*APOA1-AS*	G	A	0.719993	0.0264476	0.00223913	3.40E-32	-	-	-	No	Yes
88	rs2847500	11	120114421	*POU2F3*	G	A	0.876497	0.021925	0.00302751	4.42E-13	-	-	-	No	Yes
89	rs12317268	12	21352541	*SLCO1B1*	A	G	0.848996	0.0208967	0.00278474	6.19E-14	-	-	-	No	Yes
90	rs11182428	12	38526387	*RNA5SP358*	T	C	0.480005	0.0125352	0.00199379	3.23E-10	-	-	-	No	Yes
91	rs1038165	12	68665940	*MDM1*	T	C	0.583349	0.0120567	0.002018	2.31E-09	-	-	-	No	Yes
92	rs11108368	12	96386138	*HAL*	G	A	0.606145	0.0038307	0.00209129	0.0669916	-	-	-	No	Yes
93	rs12372115	12	97982701	*RMST*	G	T	0.929281	0.0217954	0.00387948	1.93E-08	-	-	-	No	Yes
94	rs73413596	12	111582630	*CUX2*	C	T	0.073854	0.0216996	0.00382584	1.41E-08	-	-	-	No	Yes
95	rs7149014	14	29802911	*RP11-562L8.1*	T	C	0.370807	0.0129475	0.00208615	5.42E-10	-	-	-	No	Yes
96	rs12881545	14	101176212	*DLK1*	C	G	0.673439	0.011822	0.00213142	2.91E-08	-	-	-	No	Yes
97	rs1800588	15	58723675	*ALDH1A2, LIPC*	C	T	0.784797	0.0329215	0.00242187	4.38E-42	-	-	-	No	Yes

No	SNP	CHR	BP	Gene	A1	A2	A1F	UK Biobank*			SUNLIGHT Consortium[†]			SNPs for 35 SNP genetic score	SNPs for 122 SNP genetic score
								Beta	SE	P	SNPs for 35 SNP score	SE	P		
98	rs55829990	15	63790642	*USP3*	T	C	0.655996	0.0186013	0.00210294	9.12E-19	-	-	-	No	Yes
99	rs62007299	15	77711719	*PEAK1*	G	A	0.287463	0.0133407	0.00219977	1.32E-09	-	-	-	No	Yes
100	rs325384	15	100229761	*MEF2A*	C	T	0.715795	0.0141728	0.00221789	1.66E-10	-	-	-	No	Yes
101	rs17231506	16	56994528	*CETP*	C	T	0.676894	0.0184236	0.00213148	5.45E-18	-	-	-	No	Yes
102	rs11076175	16	57006378	*CETP*	G	A	0.178358	0.0230493	0.00260705	9.47E-19	-	-	-	No	Yes
103	rs4327060	16	72807438	*RP5-991G20.1*	C	T	0.945604	0.0243589	0.00439221	2.92E-08	-	-	-	No	Yes
104	rs4575545	16	79755446	*RP11-345M22.1, RP11-345M22.2*	G	A	0.695172	0.0155823	0.00217312	7.47E-13	-	-	-	No	Yes
105	rs11542462	16	82033810	*SDR42E1*	G	A	0.865656	0.023334	0.00291776	1.27E-15	-	-	-	No	Yes
106	rs10454087	17	40735641	*RETREG3 (FAM134C)*	C	T	0.715178	0.0135306	0.00220667	8.70E-10	-	-	-	No	Yes
107	rs2952289	17	66464414	*RP11-120M18.2*	T	C	0.798032	0.017715	0.00249217	1.18E-12	-	-	-	No	Yes
108	rs8091117	18	28919794	*DSG1*	C	A	0.934702	0.0263626	0.0040284	5.98E-11	-	-	-	No	Yes
109	rs4121823	18	47144223	*LIPG*	T	A	0.154667	0.0192879	0.00277797	3.83E-12	-	-	-	No	Yes
110	rs590215	18	57904088	*RP11-795H16.2*	C	T	0.734073	0.0129217	0.00225753	1.04E-08	-	-	-	No	Yes
111	rs2037511	18	61366207	*SERPINB11*	A	G	0.166007	0.0181228	0.00267963	1.35E-11	-	-	-	No	Yes
112	rs142158911	19	11190534	*LDLR*	A	G	0.114608	0.0255317	0.00314553	4.79E-16	-	-	-	No	Yes
113	rs187429064	19	19380513	*AC138430.4, TM6SF2*	G	A	0.011266	0.0648324	0.00947915	7.95E-12	-	-	-	No	Yes
114	rs3814995	19	36342212	*NPHS1*	C	T	0.688405	0.012558	0.00214992	5.18E-09	-	-	-	No	Yes
115	rs7412	19	45412079	*APOE*	T	C	0.082073	0.0300485	0.00363434	1.36E-16	-	-	-	No	Yes
116	rs484195	19	45421877	*APOC1*	A	G	0.384386	0.0155156	0.00209699	1.37E-13	-	-	-	No	Yes
117	rs8113404	19	53065579	*ZNF808, ZNF701*	T	C	0.304586	0.012173	0.00217136	2.07E-08	-	-	-	No	Yes
118	rs11606	19	54658102	*CNOT3*	G	C	0.425162	0.0120363	0.00205119	4.41E-09	-	-	-	No	Yes
119	rs2207132	20	39142516	*MAFB*	G	A	0.96711	0.0345955	0.00557773	5.56E-10	-	-	-	No	Yes
120	rs2229742	21	16339172	*NRIP1*	G	C	0.896549	0.0251483	0.00327069	1.48E-14	-	-	-	No	Yes
121	rs6003456	22	23356100	*AP000362.1*	T	A	0.765336	0.013277	0.00236378	1.95E-08	-	-	-	No	Yes
122	rs115621755	22	50853134	*PPP6R2*	C	T	0.67288	0.0124309	0.00212296	4.76E-09	-	-	-	No	Yes

A1: serum-25(OH)D-increasing allele; A2: alternative allele; A1F: allele frequency for A1; SE: standard error; SNP: single nucleotide polymorphism; CHR: chromosome number; BP: base-pair position, Genome Reference Consortium Human Build 37 (GRCh37); Vitamin D Genetic Score: genetic instrument (using 35 GWAS variants) for measured 25(OH)D concentration used in the primary analysis; 122 version of the Vitamin D Genetic Score: genetic instrument (using 122 GWAS variants) for measured 25(OH)D concentration used in the sensitivity analysis; *obtained from Revez, 2020 et al.; [†]imputed summary statistics, obtained from Revez, 2020 et al., serum 25(OH)D has been natural-log transformed; [a]SNP proxy in the SUNLIGHT consortium: rs1123571, $r^2 = 0.86806$ (1000 Genome, EUR); [b]SNP proxy in the SUNLIGHT consortium: rs2186777, $r^2 = 1$ (1000 Genome, EUR).

Supplementary Table 1 Reference:

Revez, JA, Lin, T, Qiao, Z, Xue, A, Holtz, Y, Zhu, Z, Zeng, J, Wang, H, Sidorenko, J, Kemper, KE, Vinkhuyzen, AAE, Frater, J, Eyles, D, Burne, THJ, Mitchell, B, Martin, NG, Zhu, G, Visscher, PM, Yang, J, Wray, NR & McGrath, JJ 2020, 'Genome-wide association study identifies 143 loci associated with 25 hydroxyvitamin D concentration', *Nature Communications,* vol. 11, no. 1, p. 1647.

Table S2. Association of the vitamin D genetic score with potential confounders in the UK Biobank.

		Vitamin D-GS
	N (%)	Mean (S.D.)
BMI		
Lowest 25% - 12.1-24.1	76,640 (24.9)	38.41 (5.87)
Middle 50% - 24.1-29.8	153,321 (50.0)	38.42 (5.88)
Highest 25% - 29.8-74.7	76,675 (25.0)	38.45 (5.86)
P[1]		0.88
Smoking		
Non-smokers	167,703 (54.7)	38.42 (5.86)
Ex-smokers	108,118 (35.3)	38.44 (5.89)
Current smokers	30,719 (10.0)	38.39 (5.89)
P[1]		0.18
Alcohol intake		
Daily	65,542 (21.3)	38.34 (5.88)
1 to 4 times week	155,608 (50.6)	38.45 (5.87)
1 to 3 times month	34,098 (11.1)	38.39 (5.87)
Special occasion	32,179 (10.5)	38.36 (5.90)
Never	19,963 (6.5)	38.51 (5.85)
P[1]		0.02
Physical activity		
Low	92,012 (30.6)	38.44 (5.87)
Moderate	149,205 (49.6)	38.42 (5.88)
High	59,561 (19.8)	38.42 (5.87)
P[1]		0.67
Education		
None	52,193 (17.1)	38.44 (5.86)
NVQ/CSE/A-levels	109,099 (35.8)	38.44 (5.88)
Degree/professional	143,735 (47.1)	38.42 (5.88)
P[1]		0.55
Townsend deprivation index quartiles		
Q1 lowest	76,793 (25.0)	38.41 (5.86)
Q2	76,821 (25.0)	38.46 (5.89)
Q3	76,815 (25.0)	38.42 (5.87)
Q4 highest	76,811 (25.0)	38.43 (5.88)
P[1]		0.38

NVQ, National Vocational Qualification; CSE, Certificate of Secondary Education; A-levels, Advanced levels; SD, standard deviation; Q, quartiles. Threshold for statistical significance = 0.05/6(confounders) = 0.008. P[1] Values have been adjusted for age, sex, genotyping array, birth location, and assessment center. Vitamin D-GS: vitamin D genetic score

Table S3. Mendelian randomization analysis for the association of genetically predicted 25(OH)D with falls concurring with C-reactive protein ≥ 20 mg/L in the UK Biobank - 122 SNP genetic score and leave block out analysis.

**Vitamin D genetic score / leave-out block**	**SNPs excluded from the 35 SNP genetic score**	**Outcome* OR: 95% CIs. P**
**122 SNP Genetic score**	**N/A**	1.18: 1.02, 1.37. 0.02
**35 SNP Genetic score**	**N/A**	1.20: 1.00, 1.44. 0.05
**Blood traits block**	rs1047891, rs1352846, rs6672758, rs72997623, rs7528419,rs78151190	1.20: 0.92, 1.57. 0.18
**Lipids / Metabolic block**	rs76798800, rs78151190, rs77924615, rs261291, rs727857, rs1047891, rs12794714, rs72997623, rs7528419, rs7574138, rs6782190	1.18: 0.96, 1.44. 0.12
**Renal block**	rs1047891, rs17216707, rs77924615	1.18: 0.99, 1.42. 0.07
**Unclassified block**	rs6782190, rs61891388, rs12803256, rs1047891, rs212100, rs7522116, rs78151190, rs261291, rs12056768, rs1933064	1.20: 0.99, 1.47. 0.06
**Renal or Metabolic block**	rs76798800, rs78151190, rs77924615, rs261291, rs727857, rs1047891, rs12794714, rs72997623, rs7528419, rs75741381, rs6782190, rs77924615	1.16: 0.95, 1.42. 0.15

*Per 10 nmol/L increase in 25(OH)D. Adjustment includes age, sex, assessment center, SNP array, top 40 genetic principal components, and nuisance factors which could affect serum 25(OH)D measurements, including month in which blood sample was taken, fasting time before blood sample was taken, and sample aliquots for measurement.

Table S4. Functional blocks used in the leave-block-out analyses.

Functional block[a]	SNP	Gene	Traits
Blood traits	rs78151190	CARMIL1 (LRRC16A)	Various, incl. hemoglobin, platelet count, reticulocyte count
	rs1352846	GC	White cell count, granulocyte count
	rs1047891	CPS1	Various, incl. amino acid levels, platelet count, hemoglobin, metabolite levels
	rs72997623	DGAT2	Hemoglobin
	rs7528419	CELSR2	Blood protein levels, progranulin levels
	rs6672758	GALNT2	Platelet count, red cell distribution
Lipids/Metabolic	rs76798800	DCST2	Various, incl. fat-free mass, birth weight, height
	rs78151190	CARMIL1 (LRRC16A)	HbA1c, pulse, blood pressure
	rs77924615	PDILT	Blood pressure
	rs261291	ALDH1A2	Cholesterol, lipid metabolism
	rs727857	LINC01122	Fat mass, BMI, impedance, weight
	rs1047891	CPS1	Various, incl. weight, impedance, fat- free mass cholesterol
	rs12794714	CYP2R1	Hip circumference
	rs72997623	DGAT2	HDL cholesterol
	rs7528419	CELSR2	Angina pectoris, coronary artery disease, cholesterol, statin use
	rs75741381	VGF	Impedance
	rs6782190	CADM2	Fat free mass, BMI, impedance
Renal	rs77924615	PDILT	Glomerular filtration rate
	rs1047891	CPS1	Creatinine, chronic kidney disease
	rs17216707	CYP24A1	Creatinine, glomerular filtration rate
Unclassified	rs6782190	CADM2	Nervous feelings, alcohol intake, smoking, risk taking, number of children fathered
	rs61891388	RP11-867G23.13	Qualifications
	rs12803256	AP002387.1	Population differentiation
	rs1047891	CPS1	Headache
	rs212100	SULT2A1	Cholelithiasis, dehydroepiandrosterone sulphate
	rs7522116	FOXO6	Intelligence, qualifications
	rs78151190	CARMIL1 (LRRC16A)	Disorders of mineral metabolism
	rs261291	ALDH1A2	Age-related macular degeneration
	rs12056768	LINC00536	Hair or balding pattern: pattern 4
	rs1933064	FLG-AS1	Atopic dermatitis

[a]Functional blocks identified using trait associations identified through PhenoScanner V2[16].

Table S5. Phenotypic analyses showing the prevalence of falls across CRP ranges, and the association between falls and categorical CRP, in the UK Biobank.

CRP	Fall<br>% (cases)	Simple Model<br>OR: 95% CIs	Adjusted Model<br>OR: 95% CIs
0-0.99 mg/L	16.57 (20,167)	*Reference*	Reference
1-4.99 mg/L	20.40 (30,532)	1.23: 1.20, 1.25	1.08: 1.06, 1.11
5-9.99 mg/L	25.37 (5,649)	1.54: 1.49, 1.60	1.20: 1.16, 1.25
10-14.9 mg/L	25.77 (1,639)	1.56: 1.47, 1.66	1.18: 1.10, 1.25
15-19.9 mg/L	27.19 (741)	1.70: 1.56, 1.87	1.29: 1.17, 1.41
≥ 20 mg/L	26.72 (940)	1.67: 1.54, 1.81	1.36: 1.25, 1.48

CRP = C-reactive protein. OR = odd ratio. CI = confidence intervals. Simple models were adjusted for sex, age, assessment center, and nuisance factors that could affect 25(OH)D serum measurements, including month in which blood sample was taken, fasting time before blood sample was taken, and sample aliquot for measurement; fully adjusted models were additionally adjusted for educational status, Townsend deprivation index, body mass index, physical activity, alcohol, and smoking.

Table S6. Fully adjusted association between continuous 25(OH)D and falls, stratified above and below CRP thresholds.

CRP	OR: 95% CI. P	Cases / Controls
≥ 5 mg/L	0.9970: 0.996, 0.999. < 0.001	8,157 / 24,547
< 5 mg/L	0.9987: 0.998, 0.999. < 0.001	48,138 / 214,100
≥ 10 mg/L	0.9970: 0.995, 0.999. 0.005	2,972 / 8,739
< 10 mg/L	0.9990: 0.998, 0.999. < 0.001	53,323 / 229,908
≥ 15 mg/L	0.9980: 0.994, 1.000. 0.078	1,506 / 4,291
< 15 mg/L	0.9985, 0.998, 0.999. < 0.001	54,789 / 234,356
≥ 20 mg/L	1.0000: 0.996, 1.004. 0.723	838 / 2421
< 20 mg/L	0.9980: 0.998, 0.999. < 0.001	55,457 / 236,226

CRP = C-reactive protein. OR = odds ratio. CI = confidence intervals. Fully adjusted model was adjusted for sex, age, assessment center, and nuisance factors that could affect 25(OH)D serum measurements, including month in which blood sample was taken, fasting time before blood sample was taken, and sample aliquots for measurement; model was additionally adjusted for educational status, Townsend deprivation index, body mass index, physical activity, alcohol, and smoking.

Table S7. Fully adjusted association between continuous 25(OH)D and falls, with the outcome of falls concurring with inflammation across increasing CRP thresholds.

CRP	OR: 95% CI. P	Cases / Controls
$\geq$ 5 mg/L	0.98: 0.96, 0.99. < 0.001	8,157 / 239,322
$\geq$ 10 mg/L	0.99: 0.97, 1.01. 0.27	2,972 / 239,322
$\geq$ 15 mg/L	1.00: 0.97, 1.03. 0.91	1,506 / 239,322
$\geq$ 20 mg/L	1.04: 1.001, 1.08. 0.04	838 / 239,322

*Per 10 nmol/L increase in 25(OH)D. CRP = C-reactive protein. OR = odds ratio. CI = confidence intervals. Fully adjusted model was adjusted for sex, age, assessment center, and nuisance factors that could affect 25(OH)D serum measurements, including month in which blood sample was taken, fasting time before blood sample was taken, and sample aliquots for measurement; model was additionally adjusted for educational status, Townsend deprivation index, body mass index, physical activity, alcohol, and smoking.

Table S8. Phenotypic sensitivity analyses for the association of categorical 25(OH)D and falls in the UK Biobank, with the outcome of *falls concurring to those with rheumatoid arthritis* (adjusted and simple models).

Fall type	10-24.9 nmol/L<br>OR: 95% CI. P	25-49.9 nmol/L<br>OR: 95% CI. P	Reference	75-99.99 nmol/L<br>OR: 95% CI. P	$\geq$ 100 nmol/L<br>OR: 95% CI. P
**Adjusted**	1.28: 1.03, 1.60. 0.03	1.05: 0.90, 1.24. 0.53	*Ref.*	1.48: 1.18, 1.86. 0.001	1.64: 1.00, 2.70. 0.05
**Simple**	1.97: 1.59, 2.44. <0.001	1.26: 1.07, 1.47. 0.005	*Ref.*	1.34: 1.07, 1.69. 0.01	1.46: 0.90, 2.40. 0.13

OR = odds ratio. CI = confidence intervals. Simple models were adjusted for sex, age, assessment center, and nuisance factors that could affect 25(OH)D serum measurements, including month in which blood sample was taken, fasting time before blood sample was taken, and sample aliquots for measurement; fully adjusted models were additionally adjusted for educational status, Townsend deprivation index, body mass index, physical activity, alcohol, and smoking.

Table S9. Phenotypic analyses for the association of categorical measured 25(OH)D and falls in the UK Biobank, with falls concurring with CRP <5mg/L.

10-24.9 nmol/L<br>OR: 95% CI. P	25-49.9 nmol/L<br>OR: 95% CI. P	Reference	75-99.99 nmol/L<br>OR: 95% CI. P	$\geq$ 100 nmol/L<br>OR: 95% CI. P
1.12: 1.08, 1.16.< *0.001*	1.03: 1.005, 1.05. *0.02*	*Ref.*	1.002: 0.97, 1.04. *0.9*	0.96: 0.89, 1.05. *0.37*

OR = odds ratio. CI = confidence intervals. Simple models were adjusted for sex, age, assessment center, and nuisance factors that could affect 25(OH)D serum measurements, including month in which blood sample was taken, fasting time before blood sample was taken, and sample aliquots for measurement; fully adjusted models were additionally adjusted for educational status, Townsend deprivation index, body mass index, physical activity, alcohol, and smoking.

Supplementary References:

1. DiaSorin. LIAISON—25 OH Vitamin D TOTAL Assay 2019.
2. Townsend, P.; Phillimore, P.; Beattie, A. *Health and Deprivation: Inequality and the North*; Routledge: London, UK, 1988.
3. Nuttall, F.Q. Body Mass Index. *Nutr. Today* **2015**, *50*, 117–128.
4. Vickers, D.; Rees, P. Creating the UK National Statistics 2001 output area classification. *J. R. Stat. Soc.* **2007**, *170*, 379–403.
5. Revez, J.A.; Lin, T.; Qiao, Z.; Xue, A.; Holtz, Y.; Zhu, Z.; et al. Genome-wide association study identifies 143 loci associated with 25 hydroxyvitamin D concentration. *Nature Commun.* **2020**, 11.
6. Jiang, X.; O'Reilly, P.F.; Aschard, H.; Hsu, Y.-H.; Richards, J.B.; Dupuis, J.; et al. Genome-wide association study in 79,366 European-ancestry individuals informs the genetic architecture of 25-hydroxyvitamin D levels. *Nature Commun*. **2018**, *9*.
7. Burgess, S.; Thompson, S.G. Use of allele scores as instrumental variables for Mendelian randomization. *Int. J. Epidemiol.* **2013**, *42*, 1134–1144.
8. Palmer, T.M.; Sterne, J.A.C.; Harbord, R.M.; Lawlor, D.A.; Sheehan, N.A.; Meng, S.; et al. Instrumental Variable Estimation of Causal Risk Ratios and Causal Odds Ratios in Mendelian Randomization Analyses. *Am. J. Epidemiology* **2011**, *173*, 1392–1403.
9. Burgess, S.; Davies, N.M.; Thompson, S.G. Bias due to participant overlap in two-sample Mendelian randomization. *Genet Epidemiol.* **2016**, *40*, 597–608.
10. Cole, S.R.; Platt, R.W.; Schisterman, E.F.; Chu, H.; Westreich, D.; Richardson, D.; et al. Illustrating bias due to conditioning on a collider. *Int. J. Epidemiol.* **2010**, *39*, 417–420.
11. Dudbridge, F.; Allen, R.J.; Sheehan, N.A.; Schmidt, A.F.; Lee, J.C.; Jenkins, R.G.; et al. Adjustment for index event bias in genome-wide association studies of subsequent events. *Nature Commun.* **2019**, *10*, 1561.
12. Burgess, S.; Davies, N.M.; Thompson, S.G. Bias due to participant overlap in two-sample Mendelian randomization. *Genet. Epidemiol.* **2016**, *40*, 597–608.
13. Burgess, S.; Butterworth, A.; Thompson, S.G. Mendelian randomization analysis with multiple genetic variants using summarized data. *Genet. Epidemiol.* **2013**, *37*, 658–665.
14. Bowden, J.; Davey Smith, G.; Burgess, S. Mendelian randomization with invalid instruments: effect estimation and bias detection through Egger regression. *Int. J. Epidemiol.* **2015**, *44*, 512–525.
15. Kamat, M.A.; Blackshaw, J.A.; Young, R.; Surendran, P.; Burgess, S.; Danesh, J.; et al. PhenoScanner V2: an expanded tool for searching human genotype–phenotype associations. *Bioinformatics* **2019**, *35*, 4851–4853.
16. Burgess, S.; Davey Smith, G.; Davies, N.M.; Dudbridge, F.; Gill, D.; Glymour, M.M. et al. Guidelines for performing Mendelian randomization investigations. *Wellcome Open Res.* **2020**, *4*, 186.
